# The plasma metabolome of women in early pregnancy differs from that of non-pregnant women

**DOI:** 10.1371/journal.pone.0224682

**Published:** 2019-11-14

**Authors:** Samuel K. Handelman, Roberto Romero, Adi L. Tarca, Percy Pacora, Brian Ingram, Eli Maymon, Tinnakorn Chaiworapongsa, Sonia S. Hassan, Offer Erez

**Affiliations:** 1 Perinatology Research Branch, Division of Obstetrics and Maternal-Fetal Medicine, Division of Intramural Research, *Eunice Kennedy Shriver* National Institute of Child Health and Human Development, National Institutes of Health, U.S. Department of Health and Human Services (NICHD/NIH/DHHS), Bethesda, Maryland, and Detroit, Michigan, United States of America; 2 Department of Internal Medicine, Division of Gastroenterology and Hepatology, University of Michigan, Ann Arbor, Michigan, United States of America; 3 Department of Obstetrics and Gynecology, University of Michigan, Ann Arbor, Michigan, United States of America; 4 Department of Epidemiology and Biostatistics, Michigan State University, East Lansing, Michigan, United States of America; 5 Center for Molecular Medicine and Genetics, Wayne State University, Detroit, Michigan, United States of America; 6 Detroit Medical Center, Detroit, Michigan, United States of America; 7 Department of Obstetrics and Gynecology, Wayne State University School of Medicine, Detroit, Michigan, United States of America; 8 Department of Computer Science, Wayne State University College of Engineering, Detroit, Michigan, United States of America; 9 Metabolon Inc., Raleigh-Durham, North Carolina, United States of America; 10 Department of Obstetrics and Gynecology, Soroka University Medical Center, School of Medicine, Faculty of Health Sciences, Ben-Gurion University of the Negev, Beer-Sheva, Israel; 11 Department of Physiology, Wayne State University School of Medicine, Detroit, Michigan, United States of America; 12 Maternity Department "D," Division of Obstetrics and Gynecology, Soroka University Medical Center, School of Medicine, Faculty of Health Sciences, Ben Gurion University of the Negev, Beer-Sheva, Israel; University of Cambridge, UNITED KINGDOM

## Abstract

**Background:**

In comparison to the non-pregnant state, the first trimester of pregnancy is characterized by systemic adaptation of the mother. The extent to which these adaptive processes are reflected in the maternal blood metabolome is not well characterized.

**Objective:**

To determine the differences between the plasma metabolome of non-pregnant and pregnant women before 16 weeks gestation.

**Study design:**

This study included plasma samples from 21 non-pregnant women and 50 women with a normal pregnancy (8–16 weeks of gestation). Combined measurements by ultrahigh performance liquid chromatography/tandem mass spectrometry and by gas chromatography/mass spectrometry generated molecular abundance measurements for each sample. Molecular species detected in at least 10 samples were included in the analysis. Differential abundance was inferred based on false discovery adjusted p-values (FDR) from Mann-Whitney-Wilcoxon U tests <0.1 and a minimum median abundance ratio (fold change) of 1.5. Alternatively, metabolic data were quantile normalized to remove sample-to-sample differences in the overall metabolite abundance (adjusted analysis).

**Results:**

Overall, 637 small molecules met the inclusion criteria and were tested for association with pregnancy; 44% (281/637) of small molecules had significantly different abundance, of which 81% (229/281) were less abundant in pregnant than in non-pregnant women. Eight percent (14/169) of the metabolites that remained significant in the adjusted analysis also changed as a function of gestational age. A pathway analysis revealed enrichment in steroid metabolites related to sex hormones, caffeine metabolites, lysolipids, dipeptides, and polypeptide bradykinin derivatives (all, FDR < 0.1).

**Conclusions:**

This high-throughput mass spectrometry study identified: 1) differences between pregnant *vs*. non-pregnant women in the abundance of 44% of the profiled plasma metabolites, including known and novel molecules and pathways; and 2) specific metabolites that changed with gestational age.

## Introduction

Conception is followed by substantial adaptive maternal physiological challenges, including immune semi-allograft tolerance of the placenta [1, 2], changes in the maternal metabolism to supply nourishment and oxygen to the growing fetus [3, 4], endocrine adjustment to the presence of human chorionic gonadotropin (hCG) [5], and hCG’s effect on the maternal endocrine glands (especially the thyroid). Therefore, pregnancy is a maternal stress test, and evolution has produced maternal physiological adjustments generally sufficient to sustain pregnancy [6] and for healthy delivery at term [7–9], i.e., changes in the blood volume [10, 11], cardiovascular system [12], glomerular filtration rate [10, 13], coagulation [14–16], and maternal-fetal immune tolerance [17, 18].

To better understand the multi-system maternal physiological changes associated with pregnancy [19], high-dimensional biology approaches [20], especially appropriate in obstetrics [21, 22], may be required. In this report, metabolomics [23, 24], targeting small molecules, is used to gain a systems-level view of pregnancy-specific metabolic changes. Metabolomics has previously been used to study differences between normal [25–32] and complicated pregnancies [33, 34], preeclampsia [35–45], preterm labor or preterm delivery [22, 46–52], intrauterine growth restriction [53–58], and other outcomes [59–65].

However, the comparison between the pregnant and non-pregnant states has been less studied. Wang *et al*. [66] reported mainly Nuclear Magnetic Resonance (NMR) measurements of 87 metabolic indicators as well as cytokines (*e*.*g*. IL-18, IL-12) in pregnant and non-pregnant women. Pinto *et al*. [67] also utilized an NMR platform and measured chemical shifts in both urine and blood samples collected from pregnant and non-pregnant women and reported differences in small-molecule concentrations, i.e., branched chain amino acids and citrulline as well as macromolecules that include the same metabolic indicators as Wang *et al*. Currently, the high-throughput mass-spectrometry platform, which identifies a greater number of metabolites at lower abundances than NMR [68], has not been used to compare pregnant and non-pregnant women. Therefore, we conducted a study of metabolomes from pregnant (8–16 weeks of gestation) and non-pregnant women, using high-throughput ultrahigh performance mass-spectrometry.

## Materials and methods

A retrospective study included 21 non-pregnant women and 50 pregnant women. Plasma samples were collected from pregnant women between 8 and 16 weeks of gestation and from non-pregnant women at recruitment. All 50 pregnant women were recruited into research protocols of the Perinatology Research Branch, an intramural division of the *Eunice Kennedy Shriver* National Institute of Child Health and Human Development (NICHD), National Institutes of Health (NIH), U.S. Department of Health and Human Services (DHHS) (Bethesda, Maryland, and Detroit, Michigan) and Wayne State University (Detroit, Michigan) from the patient population at Hutzel Women’s Hospital of the Detroit Medical Center (Detroit, Michigan), described elsewhere [69, 70]. Written informed consent was obtained from all women prior to sample collection. The protocols were approved by the Human Investigation Committee of Wayne State University (IRB No. 110605MP4F) and by the Institutional Review Board of NICHD (Protocol No. OH 97-CH-N067).

All pregnant patients had a singleton gestation delivered at term (37–42 weeks of gestation), an appropriate-for-gestational-age neonate (birthweight between the 10^th^ and 90^th^ percentiles [71]), and a normal pregnancy outcome. The samples for this study were stored (immediately after collection [72]) in the Bank of Biological Materials of Wayne State University, the Detroit Medical Center, and the Perinatology Research Branch. Smoking status, age, and race were obtained by self-report. Hyperemesis gravidarum was ascertained by expert chart review.

Clinical and demographic characteristics of the study population were summarized as median and interquartile ranges (IQR) or as percentages.

### Specimen collection and storage

Blood samples were collected into tubes containing EDTA during routine care. Samples were then spun down at 1,300g and separated from packed red blood cells. Aliquots were stored below −70°C.

### Metabolomics technique

The metabolic profiling approach combined four platforms: ultrahigh performance liquid chromatography/tandem mass spectrometry (UHPLC/MS/MS) optimized for basic species, UHPLC/MS/MS optimized for acidic species, UHPLC/MS/MS optimized for uncharged polar species, and gas chromatography/mass spectrometry (GC/MS) most suitable for volatile organic molecules such as sugars. S2 Table gives the platform used to detect each compound in the PLATFORM column. Samples from pregnant women and non-pregnant women were randomized across platform run days.

Samples were processed according to previously described protocols [73, 74]; for each sample, a total of 100μL of plasma was analyzed. Using an automated liquid handler (Hamilton LabStar, Salt Lake City, UT), protein was precipitated with methanol that contained standards to report on extraction efficiency. The resulting supernatant was split into five aliquots for analysis on the four platforms, with one aliquot retained as a spare. Aliquots, dried under nitrogen and vacuum-desiccated, were subsequently reconstituted in 50μL of 0.1% formic acid in water (acidic conditions) or in 50μL of 6.5mM ammonium bicarbonate in water, under pH 8 (basic) conditions for the UHPLC/MS/MS analysis or derivatized to a final volume of 50μL for GC/MS analysis using equal parts of bistrimethyl-silyl-trifluoroacetamide and a solvent mixture of acetonitrile:dichloromethane:cyclohexane (5:4:1) with 5% triethylamine at 60°C for one hour. In addition, three types of controls were analyzed in concert with the experimental samples: aliquots of a “client matrix,” formed by pooling a small amount of each sample, served as technical replicates throughout the data set; extracted water samples served as process blanks; and a mixture of standards was spiked into every analyzed sample.

For UHPLC/MS/MS analysis, aliquots were separated using a Waters Acquity UPLC (Waters, Millford, MA) and analyzed using a Q-Exactive high resolution/accurate mass spectrometer (Thermo Fisher Scientific, Inc., Waltham, MA), which consisted of an electrospray ionization (ESI) source and an Orbitrap mass analyzer. Derivatized samples for GC/MS were separated on a 5% phenyldimethyl silicone column with helium as the carrier gas and a temperature ramp from 60°C to 340°C and then analyzed on a Thermo-Finnigan Trace DSQ MS (Thermo Fisher Scientific, Inc.) operated at unit mass resolving power with electron impact ionization and a 50–750 atomic mass unit scan range.

Metabolites were identified by automated comparison of the ion features in the experimental samples to a reference library of chemical standard entries that included retention time, molecular weight (*m/z*), preferred adducts, and in-source fragments as well as associated MS spectra; these were curated by visual inspection for quality control using software developed at Metabolon (Metabolon Inc., Research Triangle Park, NC, USA) [75]. Total ion count data, across the sampling interval of each metabolite (corresponding to area under the peak in HPLC alone), were used as a surrogate for metabolite abundance.

### Data processing

Analyte abundance on each run day was scaled so that the median total ion count, for each metabolite, would be equal across all run days. Analytes were excluded from the data set if detected in fewer than 10 samples (50% of the number of samples in the smaller group, non-pregnant). When an analyte was not detected in a given sample, this was interpreted as an abundance below the limit of detection, and the missing analyte abundance was imputed to 99% of the minimum detected total ion counts [76]; this imputation was carried out after scaling to the common median. These data are referred to as “abundance” throughout this report.

Using data from a set of reference samples profiled by Metabolon, Inc., the association between sample storage time and each metabolite’s abundance was evaluated by the Spearman’s correlation test. Metabolites found to change with storage time (p<0.05) were adjusted log-linearly (consistent with exponential decay of the compound) based on the rate of decay observed in the reference samples. Further, data were quantile-normalized [77], a procedure originally developed for microarray data processing, to transform the distribution of metabolite abundance so that it is the same across all samples. This transformation accounts for possible systematic dilution of metabolites. Quantile normalization was performed using the R package *preprocessCore* available from Bioconductor [78]. These data are referred to as “adjusted abundance.”

### Quality assurance and quality control

Metabolomics studies depend crucially on quality assurance and control[79]. Metabolon QC practices are described extensively elsewhere[80] and involves specialized software[81]. Several types of controls were analyzed in concert with the experimental samples: a pooled matrix sample generated by taking a small volume of each experimental sample served as a technical replicate; extracted water samples served as process blanks; and a cocktail of QC standards that were carefully chosen not to interfere with the measurement of endogenous compounds were spiked into every analyzed sample to monitor instrument performance and aid with chromatographic alignment. Instrument variability was determined by calculating the median relative standard deviation (RSD) for the standards that were added to each sample prior to injection into the mass spectrometers. Overall process variability was determined by calculating the median RSD for all endogenous metabolites (i.e., non-instrument standards) present in 100% of the pooled matrix samples. Experimental samples were randomized across the platform run with QC samples spaced evenly among the injections.

### Intra-assay reproducibility

To assess the reproducibility of the ion count measurements, an intra-assay coefficient of variation was calculated based on five replicates of one particular maternal plasma sample. The experimenters were blinded to these replicates. Only metabolites detected (*i*.*e*., not imputed) in 4/5 of the replicates were included in this analysis.

### Unsupervised data analysis and visualization

Principal component analysis was applied to log (base 2) transformed abundance data or to likewise-transformed adjusted abundance data. This allowed visualization of the relationship among samples in two dimensions via the first two principal components.

### Differential abundance analysis

Differences in metabolite abundance between pregnant and non-pregnant women were evaluated using Mann-Whitney-Wilcoxon U tests. The magnitude of differences was expressed as a fold change between the median abundance in the two groups. Metabolites were considered to change significantly with pregnancy given that 1) the magnitude of change was >1.5 fold, and 2) the FDR was <0.1. We customarily use an FDR threshold of 0.1 combined with a minimum effects size cut-off, which has shown improved cross-study reproducibility [43, 82]. Comparison between groups of women were performed 1) on the abundances, 2) on the adjusted abundances, and 3) as a sensitivity analysis on abundances in a reduced set of women (excluding five of the older, white, non-pregnant women) to decrease the chance that the results would be confounded by the women’s age and race (Table 1).

**Table 1:** Characteristics of the study population.

	Pregnant (n = 50)	Non-pregnant (n = 21)	p-value
**African-American ethnicity**	90%	52%	<4 × 10^−5^^a^
**Age**	23 [21–26]	29 [24–32]	<0.002^b^
**Smoking status (self-report)**	20%	4%	<0.16^a^
**Gestational age at sample**	12w4d [11w1d– 14w5d]	n/a	n/a

Values are given as % of total or as median [interquartile range]. Note that interquartile range differs from the full range in the study.

^a^ Fisher’s exact test

^b^ Mann-Whitney-Wilcoxon U test

### Pathway analysis

Metabolite-pathway assignments were drawn from a combination of expert review (supplied by Metabolon Inc.), KEGG [83], HMDB [84], and HumanCyc [85] databases. Enrichment of predefined pathways in metabolites associated with pregnancy status was tested using Fisher’s exact test followed by controlling the FDR at 10%.

### Clustering of metabolites

Spearman correlation coefficients among metabolites were determined, based on the adjusted abundance data of pregnant women, to identify groups of metabolites with a related biological role. Hierarchical clustering of metabolites using these Spearman correlation coefficients and the *cutree* method [86] were used to select 25 clusters of metabolites. Furthermore, Spearman correlations were used to generate networks of all metabolites in significantly overrepresented pathways. In these networks, connections between metabolites (edges) represent an absolute Spearman coefficient above 0.5. For each node in the networks, we determined the degree, defined as the number of edges connecting to the node.

Additionally, correlations among metabolites were compared to previous reports when potentially relevant to the interpretation of these results.

### Sensitivity analysis

To determine the effect of possible confounding variables between pregnant and non-pregnant women we have conducted two sub-analyses. In the first, the five oldest of the white non-pregnant participants were excluded to diminish differences in age and race between the two groups. In the second analysis, all self-reported smokers are removed, since smoking was prevalent among pregnant women. For further information, see S1 Supporting Information.

### Statistical testing

Unless otherwise specified, testing for association between metabolite abundance and covariates was performed using Spearman’s correlation.

All data analyses were conducted using the statistical programming language and environment R [75].

## Results

### Characteristics of the study population

The non-pregnant women included in this study were older (median age 29 vs 23), smoked less frequently (4% vs 20%), and represented a lower proportion of African-American ethnicity (52% vs 90%) compared to pregnant women (Table 1).

### Summary of differential metabolite abundance

A total of 637 metabolites were detected in 10 or more of the 71 blood samples analyzed. All 637 metabolites, along with intra-assay coefficients of variation (CV), fold changes, and significance p-values from Mann-Whitney-Wilcoxon U tests are given in S1 Table. Based on internal standards, the median instrument variability was below 5%; and, based on day to day variation in the client matrix abundance for endogenous compounds, the median total process variability was below 10%. Based on the blinded replicates provided, the median CV of detected metabolites was 12.7%; but, among metabolites changed by pregnancy, the highest CV was 9.4%. This reflects both a limited power to detect differences when metabolites are quantified with higher technical variability and the increased variation of metabolites affected by pregnancy.

**Fig 1:**
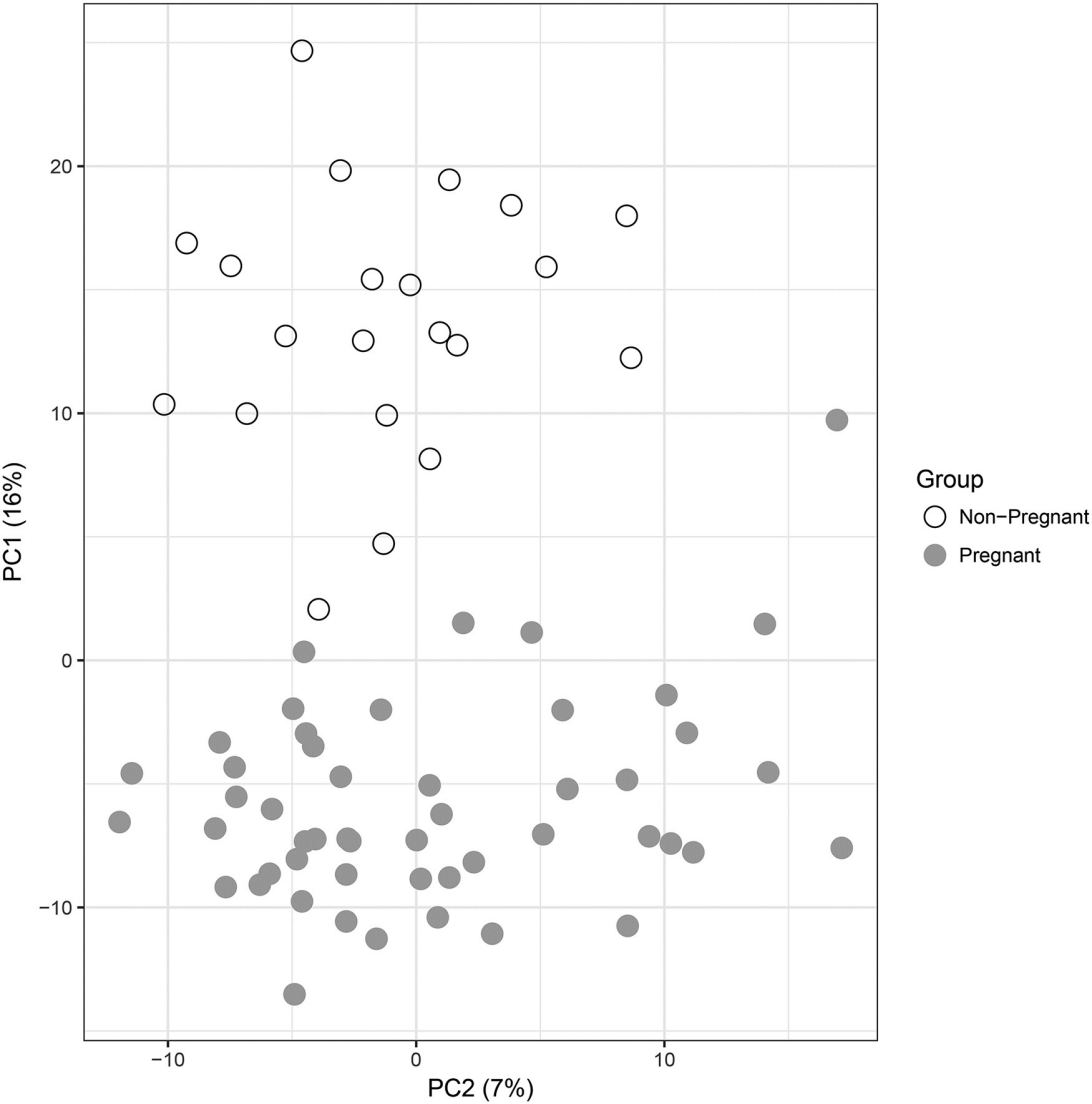
Principal component analysis of metabolic profiles of pregnant and non-pregnant women. Principal components (PC1, PC2) are derived from abundance of 637 metabolites measured in plasma samples of 50 pregnant and 21 non-pregnant women. The percentage of the variance explained by each principal component is shown in parentheses.

Pregnant and non-pregnant women differed in 281 of 637 (44%) of the metabolites profiled (FDR < 0.1 and fold change > 1.5). Were an FDR of 0.05 used without a fold-change cutoff, 368 (instead of 281) metabolites would show a significant change associated with pregnancy. In this study, the FDR threshold of 0.1 corresponded to a nominal p-value threshold of 0.05. Assuming a normal distribution of abundances in pregnant and non-pregnant women, this study is 80% powered to detect a metabolite difference between the two groups at a Cohen’s d (ratio of difference between groups to standard deviation) of 0.75 or greater at an unadjusted alpha (p-value) of 0.05. Principal components derived from the 637 metabolites showed a clear separation between the two groups of women, based on either raw abundance or adjusted metabolite abundance (Fig 1). Based on the raw abundance, 82% of significant metabolites were less abundant in pregnant than in non-pregnant women—this proportion being unlikely by chance based on a binomial test (p< 5 × 10^−28^). Both the large differences in small-molecule abundance between groups and the overall decrease in metabolite abundance with gestational age were preserved after adjustment (see S1 Supporting Information and S1 Fig).

### Individual molecule differences

**Fig 2:**
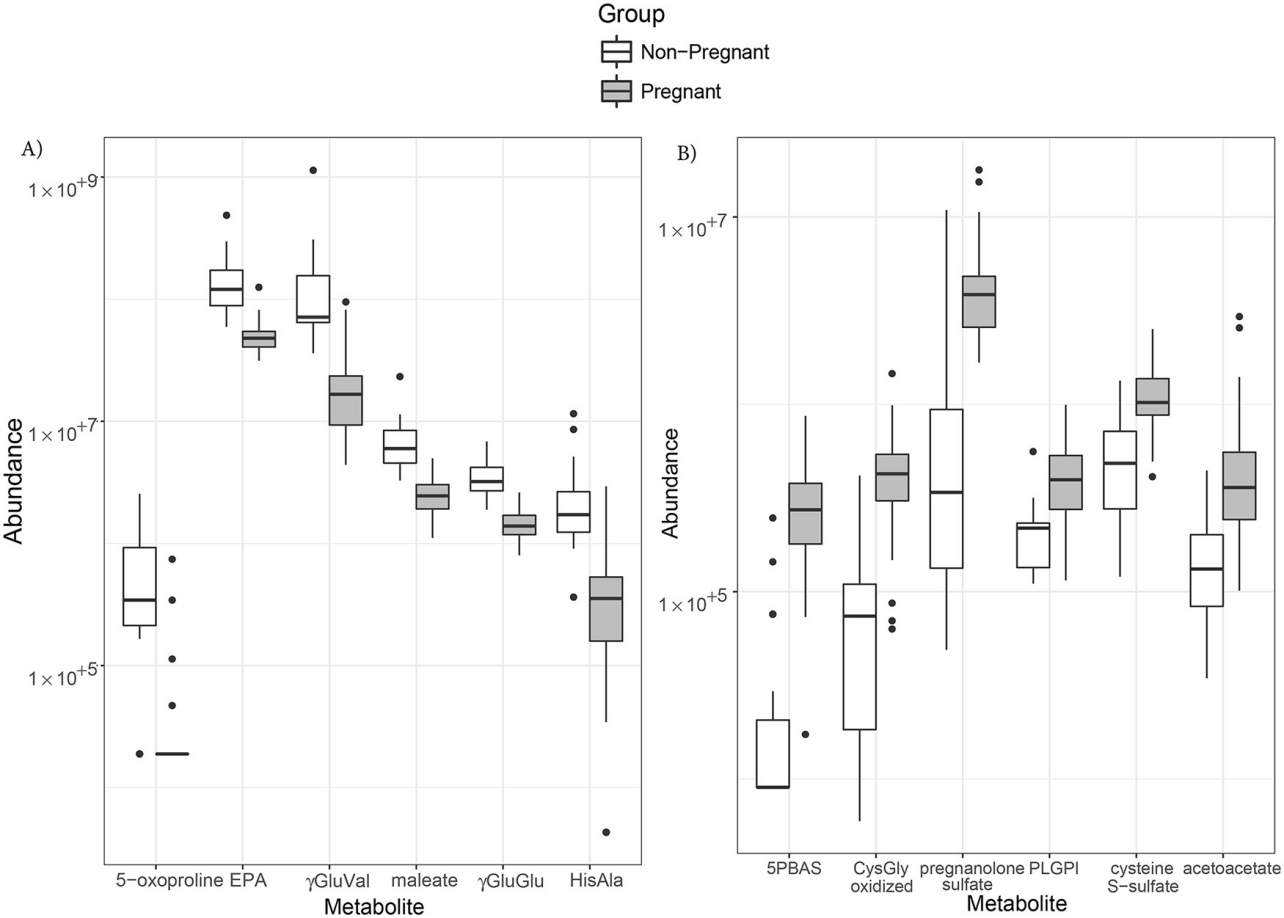
Boxplots of small-molecule abundance as a function of pregnancy status. The distribution of metabolites with increased (A) or decreased (B) abundance are shown using boxplots; the thick lines represent the medians, the boxes represent inter-quartile ranges, and the whiskers extend to the minimum/maximum values if not more than 1.5 times the interquartile range. EPA = eicosapentaenoate; γGluVal = γ-glutamyl valine; γGluGlu = γ-glutamyl glutamate; HisAla = histidylalanine; 5PBAS = 5α-pregnan-3β,20α-diol monosulfate; PLGPI = palmitoyl-linoleoyl-glycerophosphoinositol; CysGly, oxidized = glycine-cysteine-(SS)-cysteine-glycine.

Fig 2 shows box plots of abundances for the 6 most consistently decreased metabolites and for the 6 most consistently increased metabolites between groups of women, with fold-changes being reported in Table 2. Each of these metabolites belonged to a different cluster of metabolites identified based on their correlation patterns (S2 Fig).

**Table 2:** Differences among the most-consistent metabolites abundance in pregnancy.

Metabolite	Fold-change in Pregnancy	FDR
*Metabolites decreased in pregnancy*		
5-oxoproline **[87]**	0.40	3.6 × 10^−8^
eicosapentaenoate (EPA) **[88]**	0.23	3.6 × 10^−8^
gamma-glutamylvaline [89]	0.41	3.6 × 10^−8^
maleate (cis-Butenedioate) [90]	0.43	3.6 × 10^−8^
gamma-glutamylglutamate [91, 92]	0.21	8.1 × 10^−8^
histidylalanine[93]	<0.01	8.1 × 10^−8^
*Metabolites increased in pregnancy*		
5α-pregnan-3β,20α-diol monosulfate	30	6.4 × 10^−7^
cysteinyl glycine, oxidized**[94]**	5.8	1.6 × 10^−7^
allopregnanolone and pregnanolone sulfate[95]	11	2.1 × 10^−7^
palmitoyl-linoleoyl-glycerophosphoinositol[96]	1.8	1.6 × 10^−6^
cysteine s-sulfate[97]	2.1	1.8 × 10^−6^
acetoacetate**[98]**	2.7	5.6 × 10^−6^

Values are shown as fold change: ratio of median total ion count in pregnant to non-pregnant controls, followed by FDR for the corresponding Wilcoxon test. For adjusted values, refer to S1 Table. References are **bolded** when the reference indicates a previous link to obstetric outcome; other findings are believed to be novel, but a single promising reference to metabolomics and underlying physiology is provided.

Decreased metabolites included 5-oxoproline (2.5 fold); eicosapentaenoate (EPA) (4.3 fold); γ-glutamyl valine (2.4 fold); maleate (2.3 fold); γ-glutamyl glutamate (decreased 4.8 fold); and histidylalanine (>25 fold); all FDR < 8.1 × 10^−8^. In Fig 2A, given that 5-oxoproline is at or below the limit of detection/quantification in 46 of the 50 pregnant women, the boxplot appears as a plain black bar at the limit of detection.

Metabolites with increased abundance included: 5α-pregnan-3β,20α-diol monosulfate (>30 fold); oxidized cysteinylglycine (5.7 fold); allopregnanolone and pregnanolone sulfates (11.4 fold); palmitoyl-linoleoyl-glycerophosphoinositol (1.8 fold); cysteine s-sulfate (2.1 fold); and acetoacetate (2.7 fold); all FDR < 1×10^−5^. S1 Supporting Information provides a review of the studies in which these compounds were previously reported.

### Sensitivity analysis

When the five, oldest white non-pregnant controls were excluded from analysis, the observed fold change of each metabolite changed only slightly–the Spearman correlation between fold changes calculated with-and-without the five older women was 0.986 (see S1 Table for values); one of the dipeptides, γ-glutamyl lysine is among the few metabolites sensitive to this change in study population. When the self-reported smokers were excluded, the change was likewise slight, with a Spearman correlation between fold changes of 0.981. When smokers are excluded, the plasmalogen 1-stearoylplasmenylethanolamine moves below the false discovery threshold (see S1 Table).

### Pathway analysis

An over-representation analysis identified significant enrichment of five pathways: 1) xanthine metabolism, including caffeine and derivatives (13/14 metabolites significant between pregnant and non-pregnant women, FDR < 0.006); 2) steroid hormones (30/44, FDR < 0.013); 3) lysolipids (22/33, FDR < 0.06); 4) dipeptides (10/12, FDR < 0.06); and 5) polypeptides that in this study are exclusively bradykinin and derivatives (6/6, FDR < 0.06). With the exception of the steroid pathway, the small molecules in these pathways had a reduced abundance in the pregnancy group. Table 3 summarizes these findings.

**Table 3:** Pathways perturbed in pregnancy.

Pathway	Significant metabolites/ Detected metabolites	Fisher’s test p-value (FDR)	Example metabolite
**Xanthine metabolism**	13 / 14	0.0002 (0.006)	caffeine
**Steroid hormones**	30 / 44	0.0006 (0.012)	pregnanolone sulfate^a^
**Lysolipids**	22 / 33	0.0053 (0.056)	palmitoyl-linoleoyl-glycerophosphoinositol*
**Dipeptides**	10 / 12	0.0057 (0.056)	histidylalanine^a^
**Polypeptides**	6 / 6	0.0067 (0.056)	bradykinin^b^

Only the five significantly enriched (FDR < 0.1) pathways are shown.

^a^See Table 2.

^b^All 6 polypeptides detected in this study were bradykinin or derivatives thereof.

S5 Fig shows network diagrams of metabolites in each of these pathways; the edges indicate significantly correlated metabolites in pregnant women: 1) the steroid hormone network (S5 Fig **panel A**, mean degree 4.5, IQR 2–8) is split between two modules, pregnancy-increased pregnane derivatives and pregnancy-reduced androstane derivatives; 2) the lysolipid network [S5 Fig **panel B**, mean degree (number of edges to a node) 4, IQR 2–7] shows two features: a) correlated lysolipids tend to share a lysolipid head-group but not a fatty acid side-chain, and b) the network is also divided into two modules corresponding mainly to glycerophosphocholine and glycerophosphoinositol; 3) the dipeptide network (S5 Fig **panel C**, mean degree 4, IQR 2.5–5.5) demonstrates higher connectivity among dipeptides with an amino acid in common (*e*.*g*. leucylglycine (LG) is correlated with isoleucylglycine (IG), valylglycine (VG), glutamine-leucine (NL), leucylglutamine (LN), and glycylleucine (GL)). The bradykinin network contained only six nodes (S5 Fig **panel D**, mean degree 2, IQR 1.5–2 S5 Fig). The xanthine network had the highest connectivity (S5 Fig **panel E**, mean degree 7.5, IQR 6–9).

For a list of all significant correlations among metabolite abundances in pregnant women, including metabolite pairs represented in these networks, see S2 Table. Several of these correlations were previously reported in physiological studies [89, 99] of non-pregnant women (S1 Supporting Information and S3 Fig).

### Small molecules that change between 8 and 16 weeks of gestation

**Fig 3:**
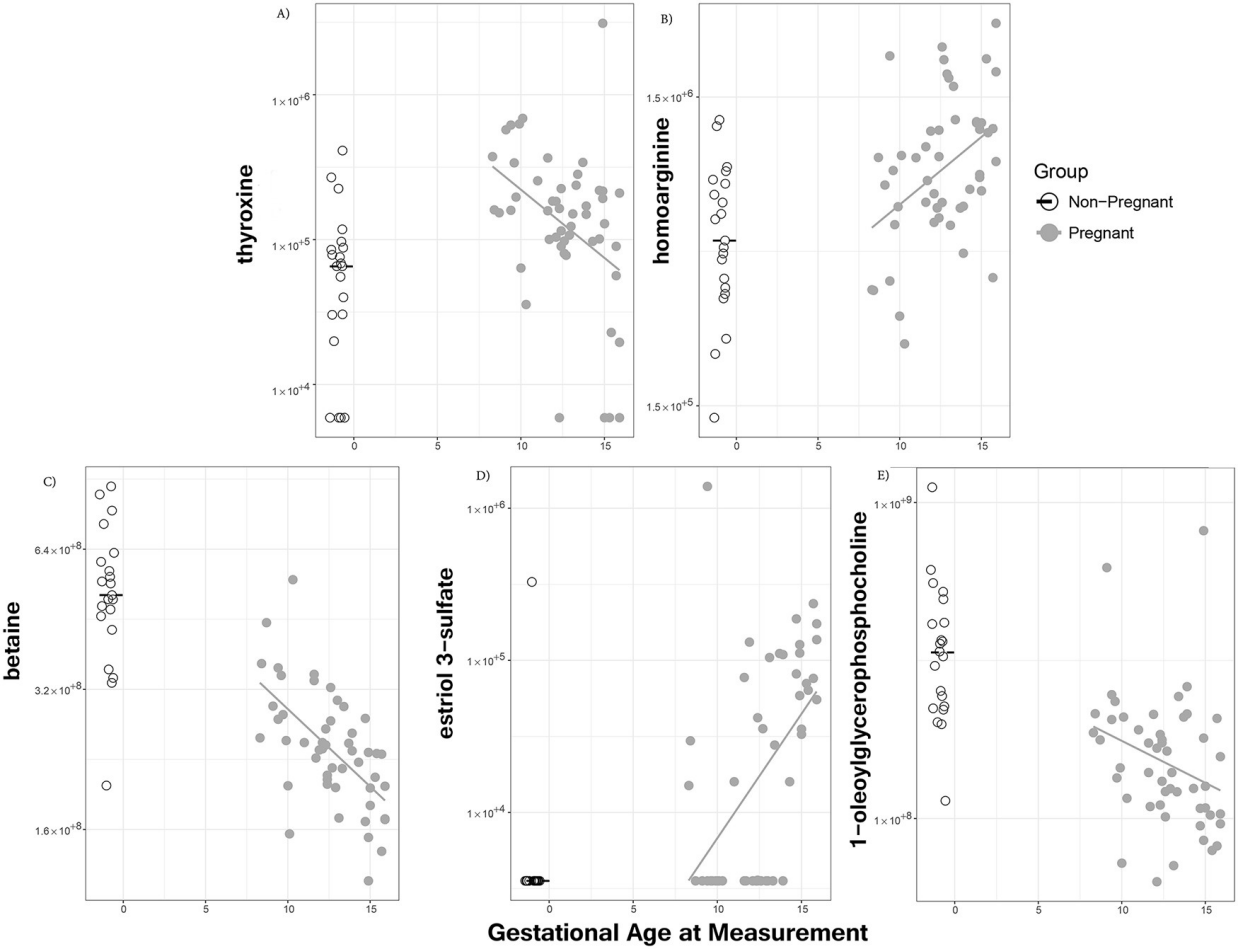
Metabolites associated with gestational age. Each panel shows metabolite abundance (on the vertical axis) *vs*. gestational age in weeks (on the horizontal axis). Non-pregnant women are shown to the left of gestational age 0 on the horizontal axis, with dither added so that points can be distinguished. Each point corresponds to one sample; linear regression lines are shown for pregnant women, and median values are shown for non-pregnant women.

Among 169 metabolites differing between pregnant and non-pregnant women, the abundance of 14 metabolites was associated with gestational age (FDR < 0.1). Of these, seven were steroids (such as estriol 3-sulfate increasing with gestational age, R = 0.57; Fig 3D) and four were lysolipids (such as 1-oleoylGPC decreasing with gestational age, R = −0.37; Fig 3E). In addition, thyroxine [100] (R = −0.41; Fig 3A), homoarginine [101–103] (R = 0.4; Fig 3B), and betaine [104] (R = −0.63, p < 9 × 10^−7^; Fig 3C) also changed with gestational age. Thyroxine was elevated (compared to non-pregnant women) at 8 weeks of gestation but declined to near non-pregnant levels by 16 weeks of gestation. Homoarginine was near non-pregnant levels at 8 weeks of gestation but then increased with gestational age while betaine was near non-pregnant levels at 8 weeks of gestation but then decreased with gestational age. With the exception of thyroxine, where gestational age dependencies and the pregnant vs non-pregnant comparison were both significant, the pattern was of pregnant/non-pregnant differences becoming more pronounced with advancing gestational age, hence supporting the differential abundance results between pregnant and non-pregnant groups.

## Discussion

### Principal findings of the study

1) Pregnant women and non-pregnant women differ in the abundance of 44% of the profiled plasma metabolites; 2) metabolite differences and associated perturbed pathways reflect physiological changes occurring in the first 16 weeks of normal pregnancy; and 3) metabolites not previously reported were identified to change as a consequence of pregnancy (e.g., blood lysolipids and dipeptides), some of which changed in accord with advancing gestation.

### What is the overall change in metabolite abundance between non-pregnancy and early pregnancy?

The abundance of nearly one-half of maternal circulating small molecules that we profiled changed within the first 16 weeks of gestation, and the majority decreased. This finding is novel and could be explained 1) by the inhibition of specific metabolic processes producing a lower abundance of small molecules [105], the activation of catabolic processes consuming small molecules (e.g. folate [44]), or 2) by expanding blood volume, leading to dilution. However, dilution effects would be removed by the transformation of the data included in the abundance adjustment. Except where compensated by increased metabolite production [45], hemodilution would affect all metabolites equally; thus, by setting the median and other quantiles of metabolite abundance to the same level across samples (quantile normalization), the direct effects of hemodilution will be cancelled out.

### Which metabolic pathways are perturbed in early pregnancy?

Five metabolic pathways were significantly enriched with metabolites differing between non-pregnant women and those in early pregnancy. These pathway perturbations provide a “system level” [75, 106] view of pregnancy. The finding of pregnancy-specific perturbations in lysolipids and dipeptides is novel. The pathways significantly perturbed with pregnancy also included steroid hormones, bradykinin derivatives, and caffeine/xanthines.

#### Steroid hormones

Of all metabolites, 5α-pregnan-3β,20α-diol monosulfate most consistently differentiated pregnant and non-pregnant women. Some changes in the abundance of the sulfated steroid hormones may result from maternal intrahepatic processes associated with pregnancy. Indeed, a ratio of sulfated to unsulfated steroid hormones in this class has been implicated in the diagnosis of intrahepatic cholestasis of pregnancy [107], although later in gestation. However, the steroid backbones of these sulfated steroids may be of placental origin, which would contribute to both gestational-age and early-pregnancy effects on steroid abundances, independent of effects in the maternal liver.

An additional group of sulfate-conjugated steroids, the pregnanolone sulfates (and allopregnanolone sulfate, an isomer), is also greatly increased in abundance for women in early pregnancy. This is in accord with previous reports [95, 108]. The role of pregnanolone during early gestation is not clear. These neuroactive steroids have been implicated in the neuro-development of the fetus [109] later in gestation. In addition, low pregnanolone isomer concentrations during gestation have been associated with subsequent post-partum depression [110], consistent with anti-anxiety GABAnergic effects of pregnanolones [111]. The lack of distinction among pregnanolone sulfate isomers is a limitation of our platform; however, targeted experiments in which these isomers are distinguished [112] validated our observation.

#### Dipeptides

The abundance of dipeptides, pairs of amino acids connected by a peptide bond, decreased in pregnant compared to non-pregnant women. A clear chemical relationship among these compounds was observed: dipeptides containing the same amino acid(s) were correlated in their abundance. This could be a consequence of either a decreased production or an increased demand of dipeptides [113]. If the latter explanation were true, then a decrease in single amino acids would be expected; however, we did not observe such a change. Therefore, the first option, a decline in dipeptide production, is more plausible. The network correlation suggests that these dipeptides are breakdown products of the same protein degradation processes (histidylalanine has been interpreted this way [93]), suggesting that protein degradation processes are lower in pregnancy.

#### Bradykinin and derivatives

All of the polypeptides measured by the platform used in this study are derivatives of bradykinin. These compounds are potent vasodilators [114] and may act synergistically with nitric oxide [115] or angiotensin [116]. The des-Arg^9^-bradykinin at the center of the network is the most active form [117]. They have a well-studied role in pregnancy-related vasodilation [118–120] during later stages of gestation.

Our observation is the first report comparing changes in bradykinin abundance between non-pregnant women and pregnant women in early gestation. A possible explanation for our observation could be derived from animal models showing that the vasodilatory effect of hCG is mediated by other mechanisms rather than through bradykinin [121] and that placental growth factor has an inhibitory effect on bradykinin activity [122].

### What are the changes in the maternal plasma metabolome between 8 and 16 weeks gestation?

There were 14 (including 7 steroids and 4 lysolipids) of 169 metabolites associated with pregnancy that also changed with gestational age. While some of these associations are novel (such as lysolipids), the increase in thyroxine abundance during the first trimester was previously reported [5, 123–126].

Steroid metabolites were the most significantly perturbed pathway in this study, and individual steroids are the most consistent changes associated with pregnancy. These observations were not surprising, given that the regulation of steroid hormones [127] is important for ovulation, conception, blastocyst implantation, and the sustenance of gestation [128–130]. This tightly regulated process involves the uterus (myometrium [131] and endometrium [132]), the cervix [133], and the ovaries [134] as well as the embryo [135, 136] and subsequently the fetal-placental unit [137]. Gestational-age changes in steroid abundance [138] over the first trimester reflect, in part, the luteal-placental shift [130]. Sulfate conjugates were the main forms of steroid hormones detected in the peripheral circulation during early pregnancy, which is in agreement with previous reports [139–141]. For example, estriol 3-sulfate increased in abundance from 10 weeks onward, resulting from production of steroid hormones by the placenta that begins in the first trimester.

Similar to steroids, lysolipids were a perturbed pathway in pregnancy, and the abundance of some lysolipids depended on gestational age. Decreasing abundance of lysolipids may reflect a physiological process by which the sensitivity of the myometrium to progesterone is enhanced. Lysophospholipids have been implicated in inhibiting the effect of progesterone and estrogen on the quiescence of the myometrium [142, 143]. Therefore, a decrease in lysophospholipid abundances could reflect a mechanism that assures the quiescence of the myometrium early in gestation, an important requirement for the maintenance of pregnancy.

In addition to potentially inter-related changes in lysolipids and hormones, amino acid derivatives also differ between pregnant and non-pregnant women. For example, the increase of homoarginine abundance with gestational age is expected in healthy pregnancies, given the role of this metabolite in vasodilation [102, 103]. Conversely, betaine, an important osmoprotectant [144] and methyl donor [145], decreases over the course of the first trimester. Our observation of declining betaine can be explained by a finding in a rat model of high placental betaine concentrations, suggesting that betaine could have been drawn from the maternal circulation as a placental methyl donor or osmoprotectant compound [146]; this observation needs further validation in human placentae. Alternatively, decreasing betaine abundance is consistent with reported low homocysteine and cysteine concentrations, inferring a lower need for methyl donation in the homocysteine pathway [147]. Mothers homozygous for the non-functional variant in betaine homocysteine s-methyl transferase (BHMT) had a 2.8-fold greater odds of placental abruption [104].

### Strengths and limitations of the study

Due to space constraints, not all of the significantly different metabolites can be discussed at length (S1 Table). The non-pregnant women in this study were somewhat older, less likely to be of African-American ethnicity, and less likely to self-report smoking (although the difference in cotinine abundances was not significant). A sensitivity analysis found no qualitative difference in the results, when older, white, non-pregnant controls were excluded, or, when self-reported smokers were excluded. Although the women in this study were not given dietary questionnaires, the great majority of differences identified have not previously been associated with dietary differences[148], and matching diets between pregnant and non-pregnant women may not be possible[149]. Finally, where gestational age differences were observed, the trend is for pregnancy-specific effects to grow, supporting that these differences are due to pregnancy; however, metabolite differences characteristic of the immediate post-implantation period of pregnancy (before 8 weeks) were not captured herein. Assessing metabolomics adaptations prior to 8 weeks of gestation will remain challenging even for future studies due to sample availability. Caffeine abundance was greatly reduced in pregnant women; this is plausibly caused by abstinence from coffee; however, we did not survey coffee consumption. In the future, a more powerful imputation approach might successfully recover additional pregnancy-specific differences[150].

In contrast to prior studies [25, 27–29, 52, 59, 61, 67], the larger sample size of this study, and the co-randomization of pregnant and non-pregnant women (which is not always done [26]) across runs of a more sensitive high-throughput mass-spectrometry metabolomic platform, enabled us to identify a larger number of metabolites associated with pregnancy.

## Conclusions

We present the first study utilizing high-throughput multi-platform chromatographic mass-spectrometry to compare the metabolite profiles of pregnant and non-pregnant women. The results of this study revealed increased pregnancy-induced maternal plasma metabolic changes, some of which corroborated previous findings. These results will have implications in further studies since metabolites with pregnancy-related changes in abundance could be prioritized for the discovery of much-needed biomarkers [82] in the “great obstetrical syndromes” [7, 151]. Moreover, efforts to identify metabolic markers of other diseases, using the same measurement platforms, must account for the metabolic effects of pregnancy: for example, lysolipids in alcoholic hepatitis [152] or dipeptides in periodontal disease [153].

## Supporting information

S1 Supporting Information This file contains text with the following subsections: “Effects of the metabolite adjustment,” “Identification of independent metabolites,” “Notes on compound names,” “Cotinine Levels,” “Reduced non-pregnant group,” “5-HEPE,” “Hyperemesis Gravidarum,” “Individual molecules,” “Abbreviations used in S5 Fig,” and “Supplementary Figure Legends”. (DOC)

S1 Table This file contains a complete list of all profiled metabolites with analysis results. (XLSX)

S2 Table This file contains pairwise associations (Spearman R values) between the adjusted abundance of individual metabolites only in the group of pregnant women. (XLSX)

S1 Fig This is a supplementary figure, “Additional plots of overall metabolites differences;” the figure legend can be viewed in S1 Supporting Information. (PDF)

S2 Fig This is a supplementary figure, “Dendrogram of all metabolite abundances;” the figure legend can be viewed in S1 Supporting Information. (PDF)

S3 Fig This is a supplementary figure, “Correlation between small molecule abundances;” the figure legend can be viewed in S1 Supporting Information. (PDF)

S4 Fig This is a supplementary figure, “Correlation between 5αpregnan3β,20β diol monosulfate abundance and body mass index (BMI);” the figure legend can be viewed in S1 Supporting Information. (PDF)

S5 Fig This is a supplementary figure, “A network representation of pathways associated with metabolic changes in pregnancy;” the figure legend can be viewed in S1 Supporting Information. (PDF)

S1 Data Set This is the complete data set used in this report. The row and column label definitions can be viewed in S1 Supporting Information.
(CSV)
