# Supplementary material for: The plasma metabolome of women in early pregnancy differs from that of non-pregnant women
Source: PLoS One. 2019 Nov 14;14(11):e0224682. doi: 10.1371/journal.pone.0224682 (PMC6855901; doi:10.1371/journal.pone.0224682)
Supplement: S1 Supporting Information — (DOC) [file pone.0224682.s001.doc]

**Supplemental Information for: The plasma metabolome in early pregnancy differs from the plasma metabolome in non-pregnant women**

Samuel K. Handelman^1,2^, Roberto Romero^1,,3,4,5,6*^, Adi L. Tarca^1,,7,8*^, Percy Pacora^1,7^, Brian Ingram^9^, Eli Maymon^1,10^, Tinnakorn Chaiworapongsa^1,7^, Sonia S. Hassan^1,7,11^, Offer Erez^1,7,12^

Note: Reference numbering in this supplement does not match reference numbering in the main text.

**Effects of the metabolite adjustment:** In normal pregnant women, there were 49 metabolites that had increased abundance, and 120 with decreased abundance in common between the adjusted and unadjusted results in comparison to non-pregnant patients.

Supplementary File 4: panel A shows boxplots of metabolite abundances in each sample as a function of pregnancy status; the reduction in mean metabolite abundance among pregnant women. Supplementary File 4: panel B shows the trend relating the per-patient median metabolite abundance to gestational age at sampling. Supplementary File 4: panel C gives adjusted boxplots (this is intended to illustrate the effect of the adjustment) and Supplementary File 4: panel D shows a comparison of the value of the first PC, *for each woman*, with and without the adjustment; the effect is minor.

**Identification of independent metabolites:** Supplementary File 5 gives a hierarchical clustering (dendrogram) of these 169 metabolites, based on adjusted measurements among pregnant women only, with color-codes corresponding to the 25 clusters. The clusters, considered as “independent” groups of metabolites, are used to choose diverse metabolites to represent in Table 2 and Figure 3.

**Note on compound names:** Names used in the text represent a compromise between precision and adherence to terminology used in established literature. If a reader is uncertain about any compound, refer to the table below which includes unique identifiers. When a number in parenthesis follows a compound name, this indicates that the compounds are one or another positional isomers.

For example:
4-androsten-3beta,17beta-diol disulfate (1)
4-androsten-3beta,17beta-diol disulfate (2)

both have the same mass and chemical formula, however, they are thought to differ in the positions in which their sulfate groups are located.

Likewise, the isomers allopregnanolone and pregnanolone (which differ only at a single chiral carbon within the backbone) are not distinguished by this platform.

**Cotinine Levels:** Pregnant women showed a median cotinine abundance of 260,000 (IQR 8,000 to 1,972,000) ion counts. Non-pregnant women showed a median cotinine abundance of 93,000 (IQR 34,000 to 1,900,000) ion counts.

Pregnant women showed a median adjusted cotinine abundance of 333,000 (IQR 14,800 to 2,422,000) ion counts. Non-pregnant women showed a median adjusted cotinine abundance of 101,000 (IQR 18,300 to 1,304,000) ion counts.

Neither difference was statistically significant.

**Sensitivity Analysis:** To assess the sensitivity of this study to differences in age and race, statistical tests were repeated with five non-pregnant women (white, aged 28, 32, 38, 43 and 46) excluded; in this reduced group, the 16 non-pregnant women are 69% black, mean age 27 (IQR 24-30) and 6% smokers (contrast with Table 1). In a second sensitivity analysis, all self-reported smokers are removed from both arms (in spite of the lack of cotinine association, noted above.) Neither change in study population affects the results in a qualitative sense (see **S1 Table** and Results section)

**5-HEPE:** 5-hydroxy-eicosapentaenoic acid (5-HEPE) was excluded because it was rarely detected (see Supplementary File 2), but the precursor EPA was detectable in reduced amounts. 5-HEPE (5-Hydroxyeicosapentaenoic acid) was not detected in the blood of *any* of the pregnant women; furthermore, it was very rarely detected in the reference samples. The basic finding is: 5-HEPE, while generally detected in most blood samples in other studies, and detected on non-pregnant controls randomized in this platform, was undetectable in the blood of the pregnant women. This may be a physiological mechanism to conserve the precursor EPA, which is depleted in the pregnancies considered in this report (see **Individual Molecules**, below.)

**Hyperemesis Gravidarum:** Two pregnant women experienced clinically-relevant nausea or vomiting within 1 week of sample collection. These women do not drive any of the reported associations and do not differ from the balance of the pregnant population in any significant way; however, some pregnancy-specific metabolite differences were not observed in these two women. The following metabolites showed a characteristic non-pregnant-like abundance in the two Hyperemesis Gravidarum patients (% gives the relative median in the two groups):

| Table: Hypermesis Gravidarum Metabolic Differences | | |
| --- | --- | --- |
| Small Molecule | Pregnant vs. Non-Pregnant | Pregnant H.E.G. vs. Pregnant not H.E.G. |
| 5α-pregnan-3α,20β-diol disulfate | 2936% | 3% |
| riboflavin (Vitamin B2) | 1851% | 14% |
| tauro-beta-muricholate | 3448% | 6% |
| 5α-androstan-3α,17β-diol monosulfate | 14% | 1691% |
| dihydroferulic acid | 14% | 2327% |
| quinate | 3% | 537% |
| stachydrine | 19% | 3340% |

**Individual molecules:** These small molecules were significantly different in their abundance, both in the unadjusted and in the adjusted ion counts. Even in this stringently defined group, 49 metabolites have increased abundance, and 120 have decreased abundance. Table 2, summarizing the 12 most consistent independent metabolites (meaning, only the single most consistent metabolite is reported from any cluster in Supplementary File 5), includes references associated with each metabolite, illustrating the breadth of the phenomena seen here. Additional literature references are provided in this section.

**Four additional small molecules increased in early pregnancy:** In addition to the metabolites discussed in the main text, the most consistent (lowest p-value) increases were seen in: 1) Oxidized cysteinyl glycine (among the oxidized thiol compounds previously associated with preeclampsia); intriguingly, oxidized cysteinyl glycine is most-strongly correlated with cyclic AMP, which is likely to be a blood pressure signal from lymphocytes. 2) A second oxidized thiol was higher in pregnancy: cysteine s-sulfate has previously been reported mainly in sulfite oxidase deficiency. 3) Increased acetoacetate is a known marker for ketonuria, which may be exacerbated by any caloric restriction during pregnancy; the observed negative association with alpha ketoglutarate would be consistent with such a metabolic shift in some pregnancies while a corresponding shift in respiratory quotient has potential as a biomarker . 4) Palmitoyl-linoleoyl-glycerophosphoinositol (PLGPI) rises in pregnancy in contrast to most lysolipids; PLGPI has been detected in a metabolomic study of prostate cancer incidence (for which pregnancy is not, of course, a risk factor).

**Four additional small molecules decreased in early pregnancy:** 1) 5-oxoproline, which was detected but at very low amounts in pregnant women; this metabolite is involved in the turnover of glutamate into gamma-glutamyl cysteine and thus to glutathione. The accumulation of 5-oxoproline indicates a deficiency in glycine and as a result a failure to convert gamma glutamyl-cysteine to glutathione . Glutathione is important in cellular detoxification and reduction processes. In addition, high neonatal concentrations of 5-oxoproline were associated with premature delivery. These reports found a correlation between abundance/concentration of betaine and 5-oxoproline; while both betaine and 5-oxoproline differentiate pregnant from non-pregnant women in this study, 5-oxoproline levels are so low that this correlation could not be tested. 2) Xiao et al. report decreased γ-glutamyl valine has been linked to decreased physical activity, in correlation with valine and isoleucine. Although neither valine nor isoleucine are significantly different in the pregnant group (indicating that this is not a direct effect of decreased physical activity as reported), the correlations Xiao et al. reported were seen for γ-glutamyl valine with valine (See Supplementary File 6: panel A). 3) Poly-unsaturated long-chain fatty acids (PUFA) such as eicosopentoate (EPA) have been proposed as a treatment for depression during pregnancy, and a failure of maternal synthesis has been proposed as a consequence of preeclampsia which may impact offspring health; a dietary intervention involving either outcome might be especially appropriate when levels are low. 4) The observed decrease in maleate (maleic acid) is novel; it may reflect a shift in the gut microbiome , but this is speculative.

**5α-pregnan-3β,20β-diol monosulfate correlated with pre-pregnancy BMI:** One metabolite showed a significant association with maternal pre-pregnancy BMI, 5α-pregnan-3β,20β-diol monosulfate (R = -0.52, p < 0.0002). This metabolite was typically over 8-fold higher in pregnant women with BMI≈20 than in non-pregnant women. In pregnant women, 5α-pregnan-3β,20β-diol monosulfate declined by 3% per pre-pregnancy BMI unit (see Supplementary File 7).

**Physiologically informative molecule pairs:** Supplementary File 6 shows associations between selected pairs of metabolites; see below for the biological interpretations of these individual pairings. Associations between the following pairs of metabolites are novel in pregnant women, and correspond to previously-characterized physiological processes, which these results associate with the early stages of pregnancy: γ-glutamyl valine abundance is correlated with valine abundance (R = 0.37, p < 0.009, see Supplementary File 6: panel A); 5α-pregnan-3β,20α-diol monosulfate with γ-glutamyl glutamine (R = -0.33, p < 0.03, Supplementary File 6: panel B); oxidized cysteinyl glycine with cyclic AMP (R = 0.51, p < 0.0002, Supplementary File 6: panel C); and, acetoacetate with alpha ketoglutarate (R = -0.51, p < 0.0002, Supplementary File 6: panel D).

**Caffeines and Xanthines:** Finally, pregnant women in this study had lower abundances of Xanthine metabolites than non-pregnant women (Supplementary File 9). A possible explanation for this result is a reduction in maternal caffeine consumption; this assumption is supported by the structure of the Xanthine metabolite correlation network. The network is highly connected (contrast Supplementary File 9 to Figure 3) indicating a strong correlation among all of the components. If the decrease in Xanthine metabolites were due to pregnancy-specific changes in gene/protein expression, a mixture of increases and decreases would be expected: for example, while the substrate of an enzyme with increased activity would be expected to decrease, the product would be expected to increase. Exactly such a pattern has been shown in non-pregnancy studies of genes acting on the Xanthine pathway

**Abbreviations used in S8 Fig**

Panel A (Steroid Hormones):

[1] "11DHOCS = 11-dehydrocorticosterone"

[2] "16aHO3S = 16a-hydroxy DHEA 3-sulfate"

[3] "17aHOPG = 17alpha-hydroxypregnanolone glucuronide"

[4] "17aHOP = 17-alpha-hydroxyprogesterone"

[5] "20aOH2P = 20a-dihydroprogesterone"

[6] "21HOPS2 = 21-hydroxypregnenolone disulfate"

[7] "21HOPS = 21-hydroxypregnenolone monosulfate (1)"

[8] "21HOPS+ = 21-hydroxypregnenolone monosulfate (2)"

[9] "4AAAS = 4-androsten-3alpha,17alpha-diol monosulfate (2)"

[10] "4AAAS+ = 4-androsten-3alpha,17alpha-diol monosulfate (3)"

[11] "4ABBS2 = 4-androsten-3beta,17beta-diol disulfate (1)"

[12] "4ABBS2+ = 4-androsten-3beta,17beta-diol disulfate (2)"

[13] "4ABBS = 4-androsten-3beta,17beta-diol monosulfate (1)"

[14] "4ABBS+ = 4-androsten-3beta,17beta-diol monosulfate (2)"

[15] "5AAAS2 = 5alpha-androstan-3alpha,17alpha-diol disulfate"

[16] "5AAAS = 5alpha-androstan-3alpha,17alpha-diol monosulfate"

[17] "5AABS2 = 5alpha-androstan-3alpha,17beta-diol disulfate"

[18] "5AABS = 5alpha-androstan-3alpha,17beta-diol monosulfate (1)"

[19] "5AABS+ = 5alpha-androstan-3alpha,17beta-diol monosulfate (2)"

[20] "5ABAS2 = 5alpha-androstan-3beta,17alpha-diol disulfate"

[21] "5ABBS2 = 5alpha-androstan-3beta,17beta-diol disulfate"

[22] "5ABBS = 5alpha-androstan-3beta,17beta-diol monosulfate (2)"

[23] "5PXBS2 = 5alpha-pregnan-3(alpha or beta),20beta-diol disulfate"

[24] "5PABS2 = 5alpha-pregnan-3alpha,20beta-diol disulfate 1"

[25] "5PBAS2 = 5alpha-pregnan-3beta,20alpha-diol disulfate"

[26] "5PBAS = 5alpha-pregnan-3beta,20alpha-diol monosulfate (1)"

[27] "5PBAS+ = 5alpha-pregnan-3beta,20alpha-diol monosulfate (2)"

[28] "5PBBS = 5alpha-pregnan-3beta,20beta-diol monosulfate (1)"

[29] "5PBX3S = 5-pregnen-3b, 17-diol-20-one 3-sulfate"

[30] "AndS = andro steroid monosulfate (1)*"

[31] "AonS = androsterone sulfate"

[32] "Cson = corticosterone"

[33] "Col = cortisol"

[34] "Con = cortisone"

[35] "DHEA-S = dehydroisoandrosterone sulfate (DHEA-S)"

[36] "EEA-S = epiandrosterone sulfate"

[37] "EolS = estriol 3-sulfate"

[38] "EonS = estrone 3-sulfate"

[39] "EcGl = etiocholanolone glucuronide"

[40] "PrgS = pregn steroid monosulfate*"

[41] "PolGl = pregnanediol-3-glucuronide"

[42] "ApregS = pregnanolone/allopregnanolone sulfate"

[43] "P2olS2 = pregnen-diol disulfate*"

[44] "PonS = pregnenolone sulfate"

Panel B (Lysolipids):

[1] "arGPC = 1-arachidonoylglycerophosphocholine (20:4n6)*"

[2] "arGPE = 1-arachidonoylglycerophosphoethanolamine*"

[3] "arGPI = 1-arachidonoylglycerophosphoinositol*"

[4] "arGPA = 1-arachidonoylglyercophosphate"

[5] "lnGPC = 1-linolenoylglycerophosphocholine (18:3n3)*"

[6] "liGPC = 1-linoleoylglycerophosphocholine (18:2n6)"

[7] "liGPE = 1-linoleoylglycerophosphoethanolamine*"

[8] "liGPI = 1-linoleoylglycerophosphoinositol*"

[9] "olGPA = 1-oleoylglycerophosphate"

[10] "olGPC = 1-oleoylglycerophosphocholine (18:1)"

[11] "olGPE = 1-oleoylglycerophosphoethanolamine"

[12] "olGPG = 1-oleoylglycerophosphoglycerol*"

[13] "olGPI = 1-oleoylglycerophosphoinositol*"

[14] "olPmE = 1-oleoylplasmenylethanolamine*"

[15] "POGPC = 1-palmitoleoylglycerophosphocholine (16:1)*"

[16] "paGPA = 1-palmitoylglycerophosphate"

[17] "paGPC = 1-palmitoylglycerophosphocholine (16:0)"

[18] "paGPE = 1-palmitoylglycerophosphoethanolamine"

[19] "paGPG = 1-palmitoylglycerophosphoglycerol*"

[20] "paGPI = 1-palmitoylglycerophosphoinositol*"

[21] "paPmE = 1-palmitoylplasmenylethanolamine*"

[22] "stGPC = 1-stearoylglycerophosphocholine (18:0)"

[23] "stGPE = 1-stearoylglycerophosphoethanolamine"

[24] "stGPI = 1-stearoylglycerophosphoinositol"

[25] "stGPS = 1-stearoylglycerophosphoserine*"

[26] "stPmE = 1-stearoylplasmenylethanolamine*"

[27] "2POGPC = 2-palmitoleoylglycerophosphocholine*"

[28] "2paGPC = 2-palmitoylglycerophosphocholine*"

[29] "2stGPC = 2-stearoylglycerophosphocholine*"

[30] "2stGPE = 2-stearoylglycerophosphoethanolamine*"

[31] "OLGPI = oleoyl-linoleoyl-glycerophosphoinositol (1)*"

[32] "PLGPI = palmitoyl-linoleoyl-glycerophosphoinositol (1)*"

[33] "POGPG = palmitoyl-oleoyl-glycerophosphoglycerol (2)*"

Panel C (Dipeptides):
[1] "AL = alanylleucine" "NL = glutamine-leucine"

[3] "GL = glycylleucine" "HA = histidylalanine"

[5] "IG = isoleucylglycine" "LN = leucylglutamine*"

[7] "LG = leucylglycine" "FA = phenylalanylalanine"

[9] "PG = prolylglycine" "TF = threonylphenylalanine"

[11] "VG = valylglycine" "VL = valylleucine"

Panel D (Bradykinin):
[1] "bkn = bradykinin"

[2] "bkn,R9-des = bradykinin, des-arg(9)"

[3] "bkn,P3-OH = bradykinin, hydroxy-pro(3)"

[4] "_LLR = HWESASLLR"

[5] "_XX = HWESASXX*"

[6] "X_XXR = XHWESASXXR*"

Panel E (Caffeine):
[1] "tmU = 1,3,7-trimethylurate"

[2] "1,3mU = 1,3-dimethylurate"

[3] "1,7mU = 1,7-dimethylurate"

[4] "1mU = 1-methylurate"

[5] "1mX = 1-methylxanthine"

[6] "3,7mU = 3,7-dimethylurate"

[7] "3mX = 3-methylxanthine"

[8] "5*,6N,3mU = 5-acetylamino-6-amino-3-methyluracil"

[9] "5*,6*,3mU = 5-acetylamino-6-formylamino-3-methyluracil"

[10] "7mX = 7-methylxanthine"

[11] "Caffeine = caffeine"

[12] "paraX = paraxanthine"

[13] "thBr = theobromine"

[14] "thPh = theophylline"

**Supplementary Figure Legends**

**S1 Fig: Additional plots of overall metabolites differences.**

Panel A gives boxplots of metabolite abundances *in each woman*. Clear boxes are non-pregnant, shaded boxes are pregnant. Although modest, the decline in the median abundance is visible.

Panel B gives the same per-sample median as a function of gestational age; the trend is not significant, but the difference in median abundance between pregnant and non-pregnant women may be more readily visible from this figure than from Panel A.

Panel C is as per Panel A, but after adjustment: this is meant to show what the quantile normalizion does – the method forces the distribution of abundances for each woman to be identical.

Panel D compares the per-woman values of the first principal component before and after adjustment: because the values before and after adjustment are similar, the adjustment perserves not only the overall pregnant/non-pregnant difference, but the relative contribution of individual metabolites to the first principal component remains similar before and after adjustment.

**S2 Fig: Dendrogram of all metabolite abundances.** This is the form of tree that would typically accompany a heatmap; an evolutionary relationship is *not* what is being shown, only strength of correlations. The branch lengths are derived from a distance function equal to 1 – R_spearman_. The color codes correspond to 25 clusters.

**S3 Fig: Correlation between small molecule abundances**. Each panel shows abundances for a pair of metabolites (one on each axis). Each point corresponds to one sample; lines are linear regression lines. Metabolites shown differ significantly with pregnancy, with the exception of valine.

**S4 Fig: Correlation between 5αpregnan3β,20β diol monosulfate abundance and body mass index (BMI).** 5αpregnan3β,20β diol monosulfate abundance (on the vertical axis) is shown vs. pre-pregnancy BMI (on the horizontal axis). Each point corresponds to one sample; regression lines are shown.

**S5 Fig: A network representation of pathways associated with metabolic changes in pregnancy.** Each node in the graph (circle) represents a metabolite while edges (lines) represent a significant Spearman correlation among pregnant women; solid lines represent a positive correlation, dotted lines a negative correlation. Metabolites with increased abundance in pregnancy are shown in gray while those with decreased abundance are shown as empty circles, with large circles indicating significance in both adjusted and non-adjusted abundance data. Metabolites that do not demonstrate significant change in abundance are shown with black circles. Metabolites’ names are abbreviated: see **S1 Supporting Information** for the full list of abbreviations; partial guides to the abbreviations are included here. (**A**) Steroid network. 5A and 4A abbreviate 5α-androstan and 4-androsten, respectively, while 5P abbreviates 5α-pregnan, with subsequent A’s and B’s representing whether the 3 OH and 17-or-20 OH are alpha or beta, respectively. (**B**) Lysolipid network. The first two letters abbreviate the fatty acid side-chain while the last three letters abbreviate the head-group. (**C**) Dipeptide network. Standard single-letter amino acid abbreviations are used. (D) Bradykinin network. All metabolites in this network are significantly decreased in abundance in the pregnant group, whether or not the adjusted abundances are used. **(F)** Xanthine/caffeine network, the decrease across all metabolites in the pathway is consistent with reduced caffeine intake.

**S1 Data Set: Complete data set.** The first eleven columns contain information on each metabolite, in order: Metabolon Compound ID; Limit of Detection (reported by Metabolon for the corresponding runs); Halflife (likewise estimated with Metabolon based on larger samples stored in the same facility; “NA” indicates no decay was detected); Chemical Name; Super Pathway (from Metabolon); Sub Pathway (from Metabolon); Platform (see text); Pubchem ID; CAS ID; KEGG ID; and, HMDB ID. The first six rows contain information on each plasma sample, including: Gestational Age (GA, for the pregnant women only); Group (pregnant or not at time of sample collection); Age of plasma donor/mother at sample collection; Race of donor/mother (self-report); Smoking status (self-report); and Body Mass Index (BMI) at measurement visit, where available. The values in the body of the table are run-day adjusted ion counts for the corresponding metabolite and plasma sample (see text).

**References**

1. Psychogios N, Hau DD, Peng J, Guo AC, Mandal R, et al. (2011) The human serum metabolome. PloS one 6: e16957.

2. Wishart DS, Knox C, Guo AC, Eisner R, Young N, et al. (2009) HMDB: a knowledgebase for the human metabolome. Nucleic acids research 37: D603-D610.

3. Quehenberger O, Armando AM, Brown AH, Milne SB, Myers DS, et al. (2010) Lipidomics reveals a remarkable diversity of lipids in human plasma. Journal of lipid research 51: 3299-3305.

4. Raijmakers MT, Zusterzeel PL, Roes EM, Steegers EA, Mulder TP, et al. (2001) Oxidized and free whole blood thiols in preeclampsia. Obstetrics & Gynecology 97: 272-276.

5. Mills P, Dimsdale J, Ziegler M (1989) Lymphocyte basal cyclic AMP production predicts blood pressure. Clinical and Experimental Hypertension Part A: Theory and Practice 11: 521-530.

6. Olney JW, Misra CH, De Gubareff T (1975) Cysteine-S-Sulfate: Brain Damaging Metabolite in Sulfite Oxidase Deficiency1. Journal of Neuropathology & Experimental Neurology 34: 167-177.

7. Coetzee E, Jackson W, Berman P (1980) Ketonuria in pregnancy—with special reference to calorie-restricted food intake in obese diabetics. Diabetes 29: 177-181.

8. Lanza IR, Zhang S, Ward LE, Karakelides H, Raftery D, et al. (2010) Quantitative metabolomics by 1 H-NMR and LC-MS/MS confirms altered metabolic pathways in diabetes. PloS one 5: e10538.

9. Melzer K, Kayser B, Schutz Y (2014) Respiratory quotient evolution during normal pregnancy: what nutritional or clinical information can we get out of it? Eur J Obstet Gynecol Reprod Biol 176: 5-9.

10. Mondul AM, Moore SC, Weinstein SJ, Karoly ED, Sampson JN, et al. (2015) Metabolomic analysis of prostate cancer risk in a prospective cohort: The alpha‐tocolpherol, beta‐carotene cancer prevention (ATBC) study. International journal of cancer 137: 2124-2132.

11. Friesen RW, Novak EM, Hasman D, Innis SM (2007) Relationship of dimethylglycine, choline, and betaine with oxoproline in plasma of pregnant women and their newborn infants. The Journal of nutrition 137: 2641-2646.

12. de Matos DG, Gasparrini B, Pasqualini SR, Thompson JG (2002) Effect of glutathione synthesis stimulation during in vitro maturation of ovine oocytes on embryo development and intracellular peroxide content. Theriogenology 57: 1443-1451.

13. Xiao Q, Moore SC, Keadle SK, Xiang Y-B, Zheng W, et al. (2016) Objectively measured physical activity and plasma metabolomics in the Shanghai Physical Activity Study. International journal of epidemiology: dyw033.

14. Su K-P, Huang S-Y, Chiu T-H, Huang K-C, Huang C-L, et al. (2008) Omega-3 fatty acids for major depressive disorder during pregnancy: results from a randomized, double-blind, placebo-controlled trial. Journal of Clinical Psychiatry 69: 644.

15. Mackay VA, Huda SS, Stewart FM, Tham K, McKenna LA, et al. (2012) Preeclampsia Is Associated With Compromised Maternal Synthesis of Long-Chain Polyunsaturated Fatty Acids, Leading to Offspring DeficiencyNovelty and Significance. Hypertension 60: 1078-1085.

16. Nakajima-Kambe T, Nozue T, Mukouyama M, Nakahara T (1997) Bioconversion of maleic acid to fumaric acid by Pseudomonas alcaligenes strain XD-1. Journal of fermentation and bioengineering 84: 165-168.

17. Kalow W, Tang BK (1991) Use of caffeine metabolite ratios to explore CYP1A2 and xanthine oxidase activities. Clinical Pharmacology & Therapeutics 50: 508-519.
