# Supplementary figures and images for: The plasma metabolome of women in early pregnancy differs from that of non-pregnant women
Source: PLoS One. 2019 Nov 14;14(11):e0224682. doi: 10.1371/journal.pone.0224682 (PMC6855901; doi:10.1371/journal.pone.0224682)

A

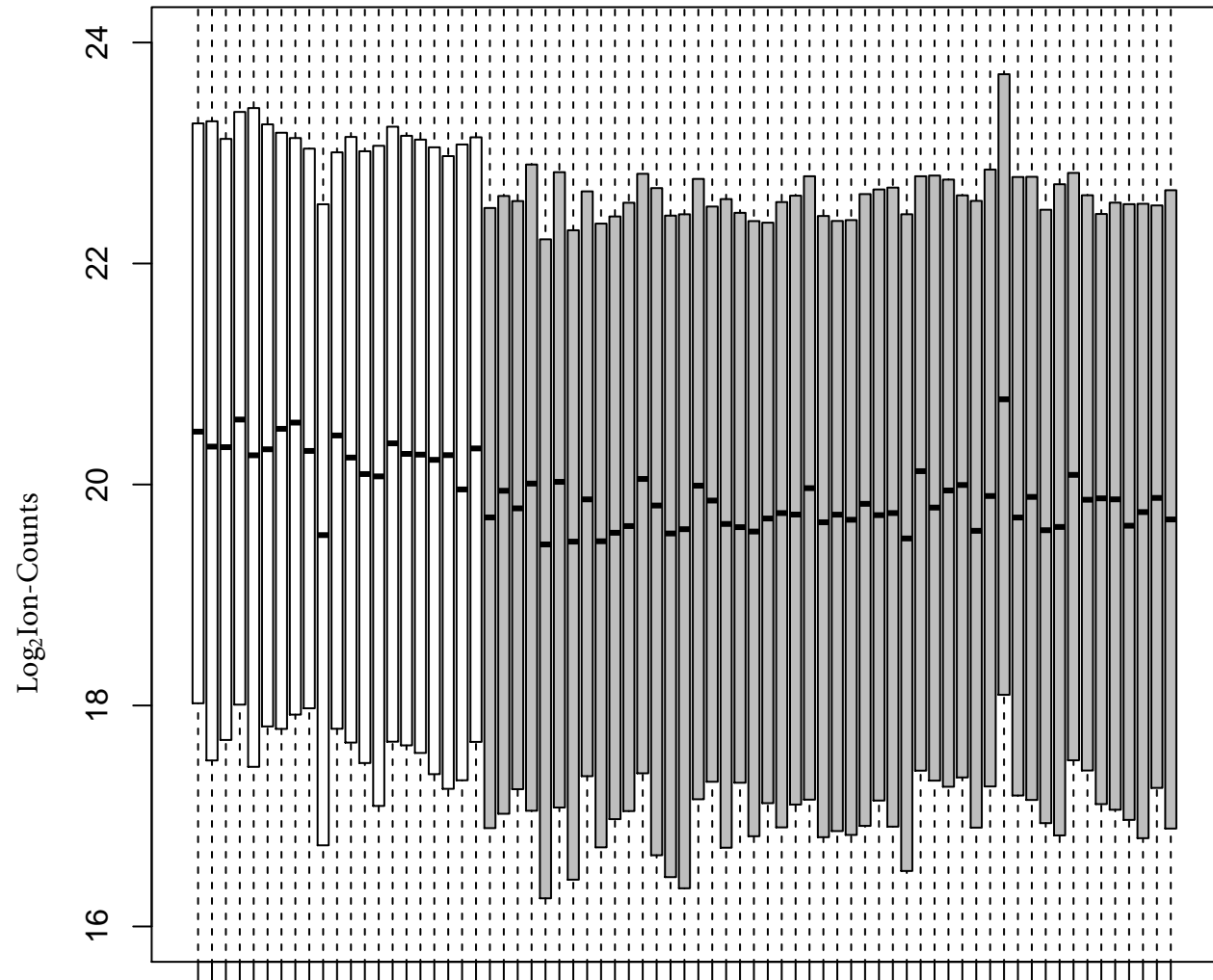

B

Individual Patient Median Metabolite Abundance

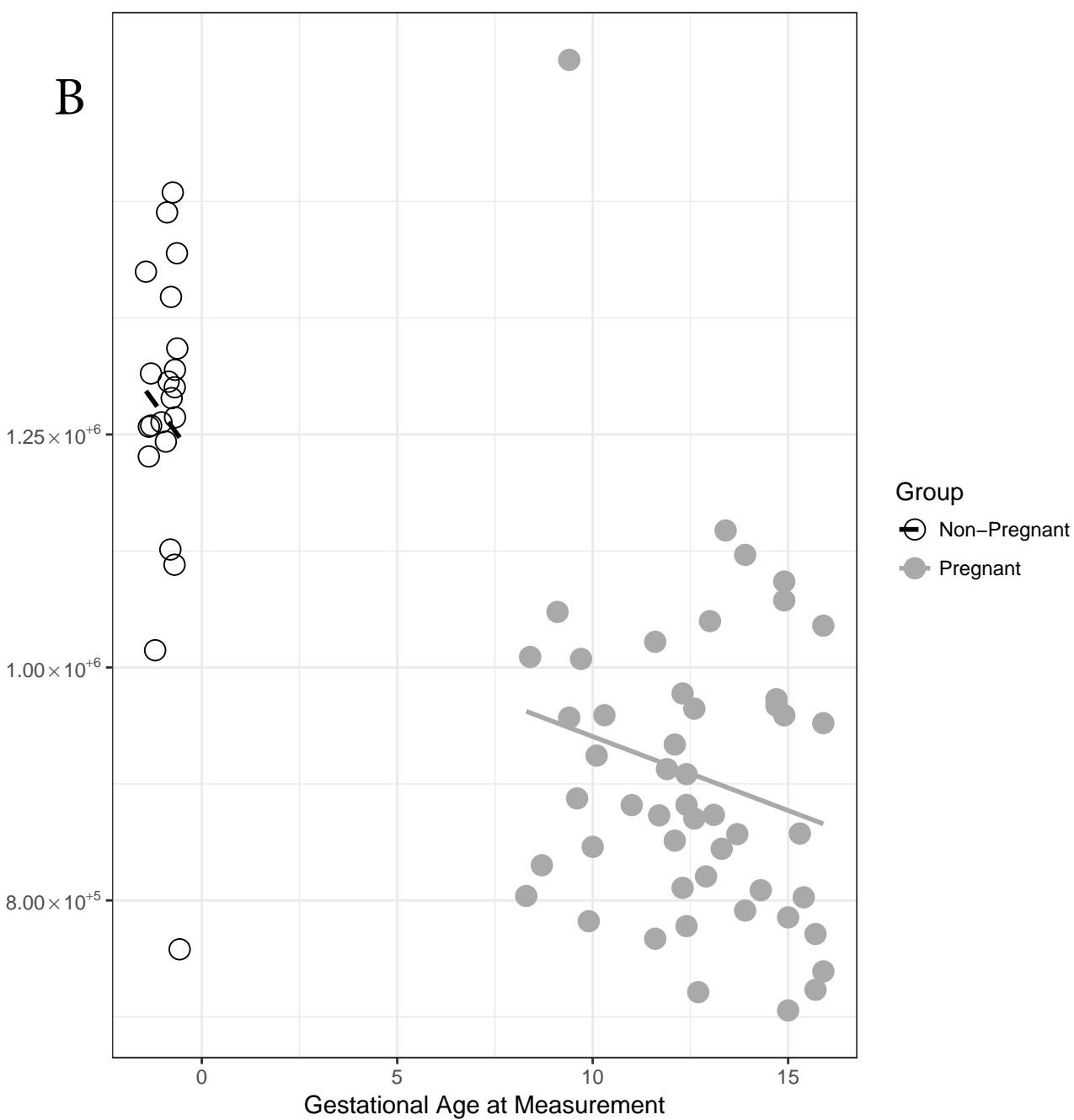

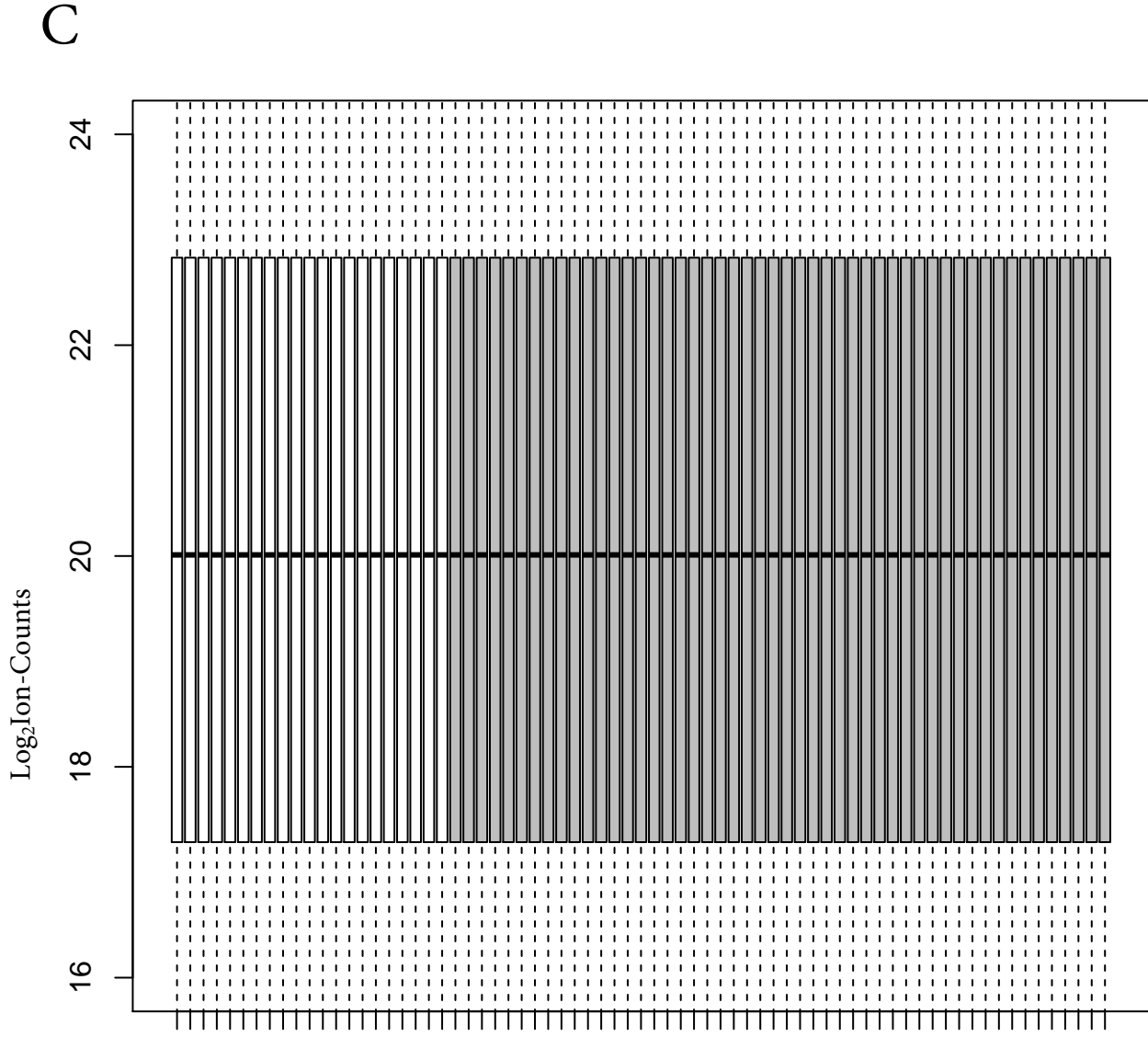

D

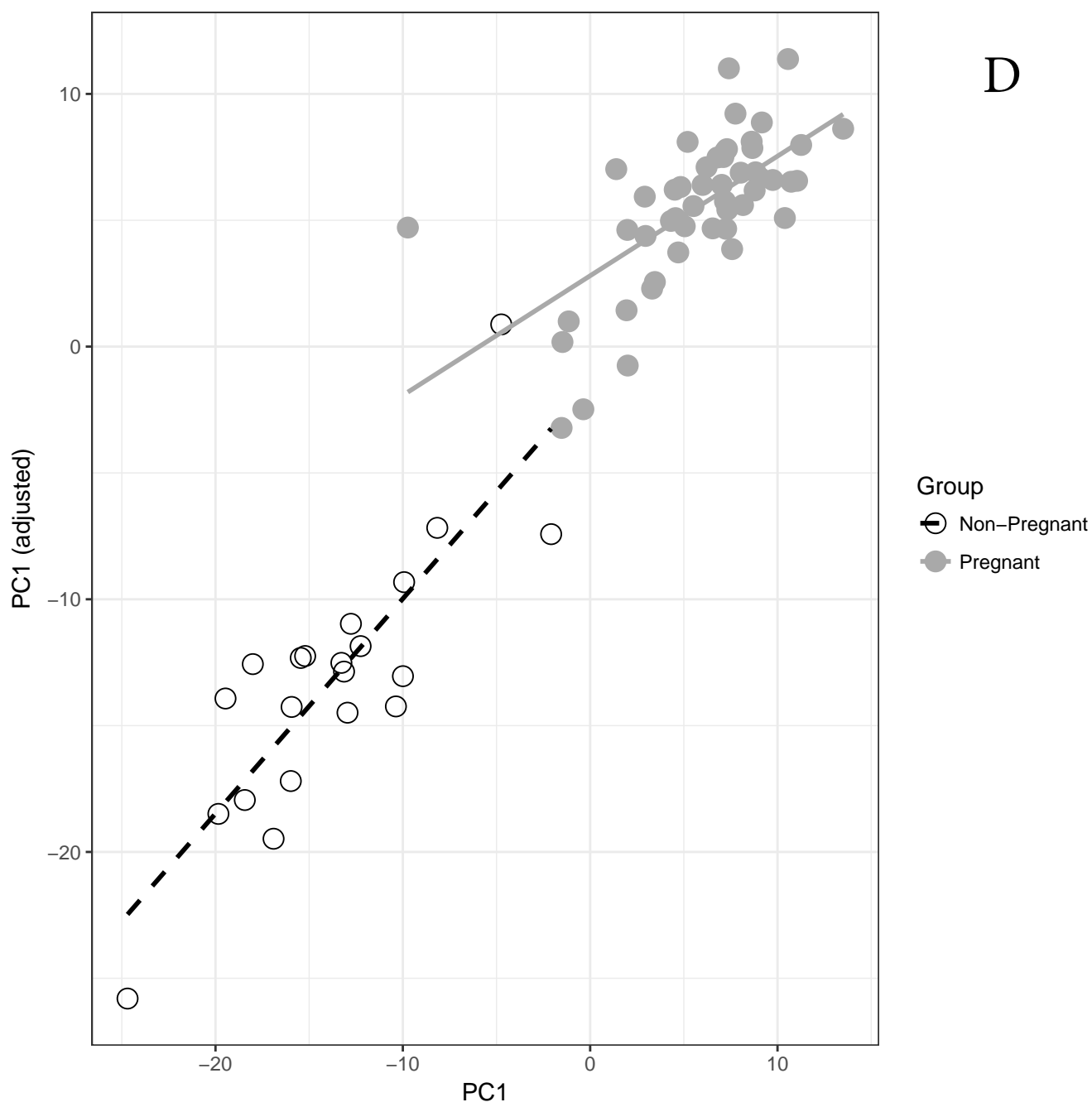

Supplement: S1 Fig — (PDF) [file pone.0224682.s004.pdf]

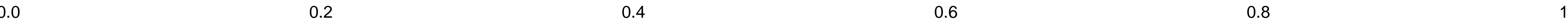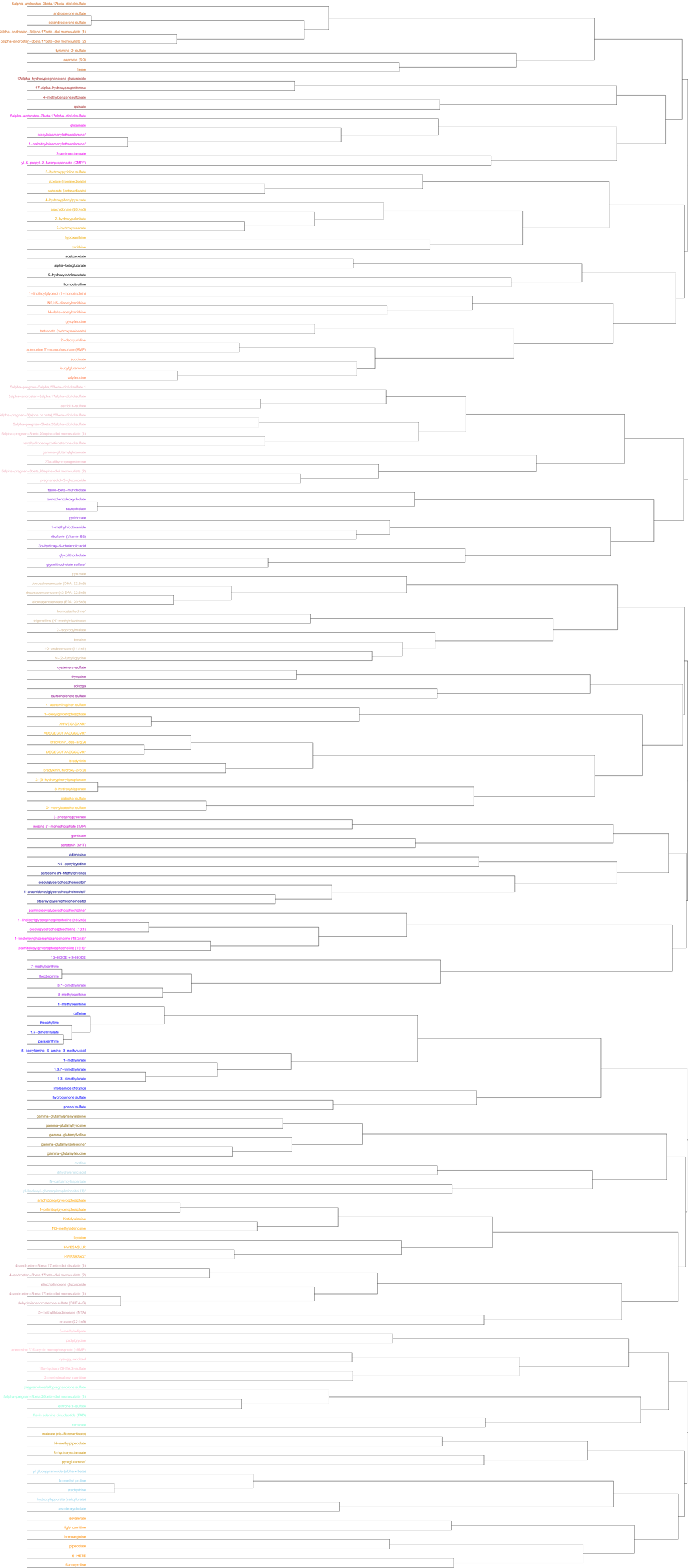

Supplement: S2 Fig — (PDF) [file pone.0224682.s005.pdf]

A

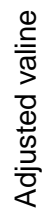

Group

☐ Non-Pregnant

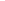 Pregnant

Adjusted  $\gamma$ GluVal

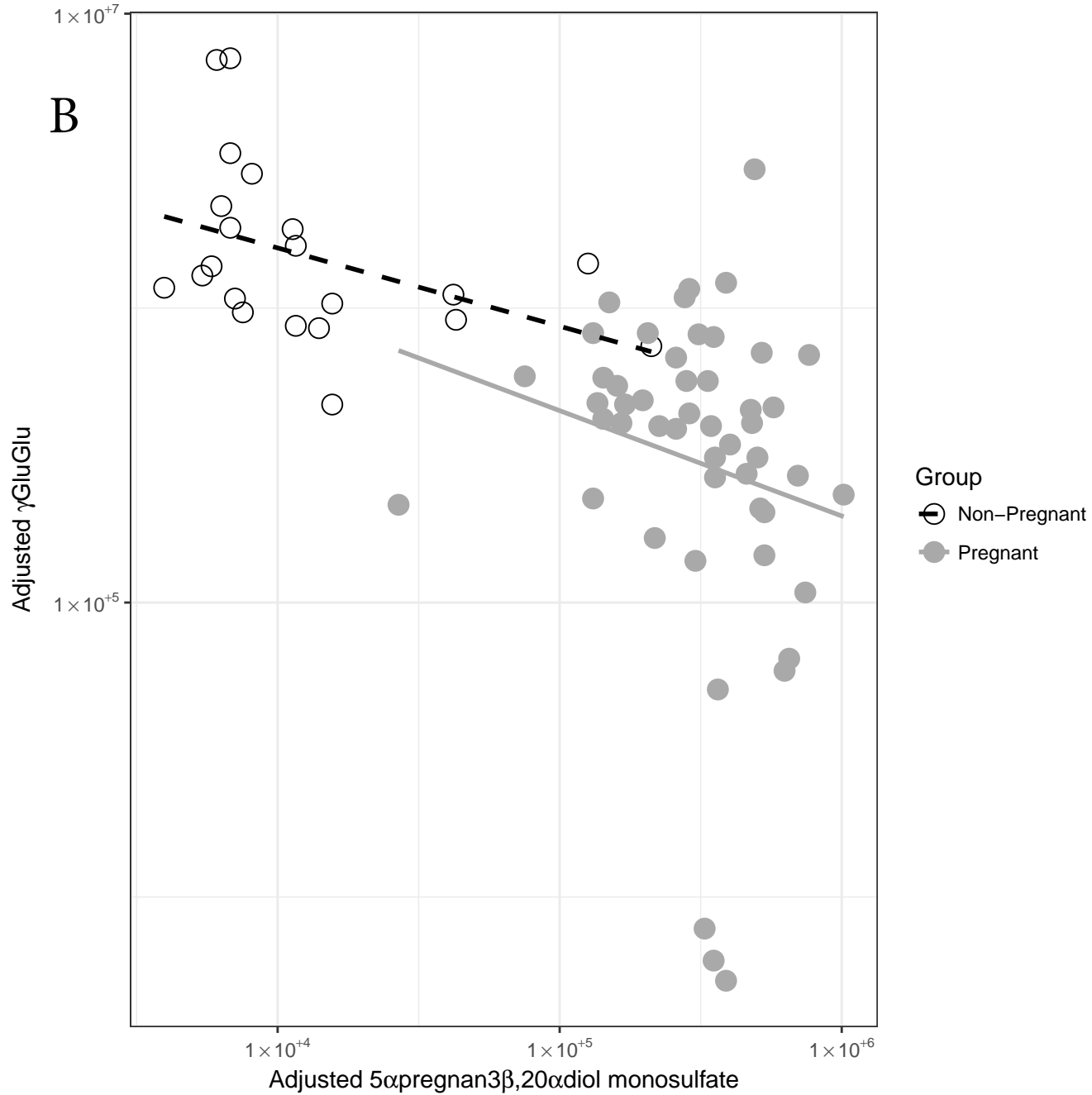

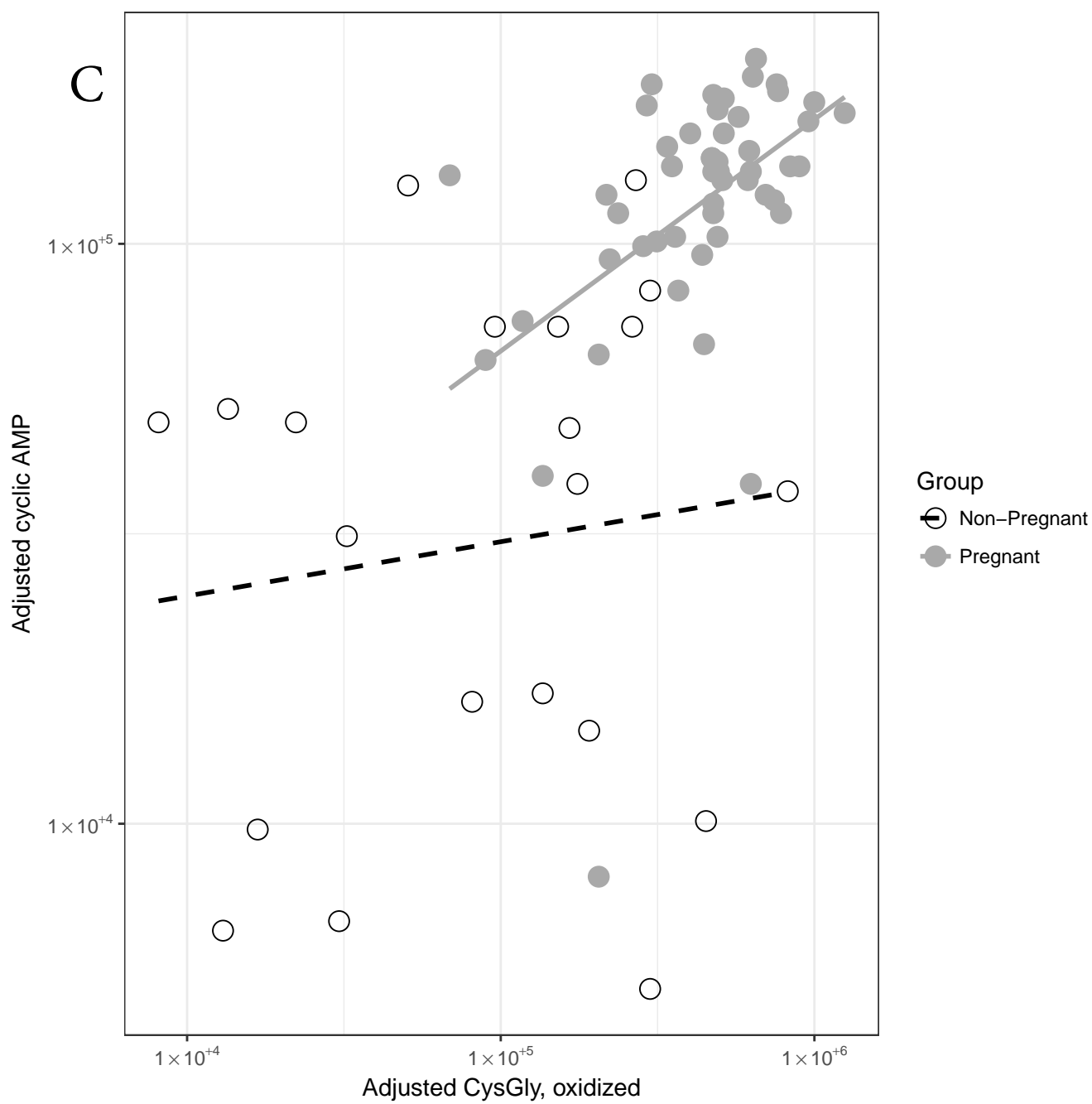

D

Adjusted alpha-ketoglutarate

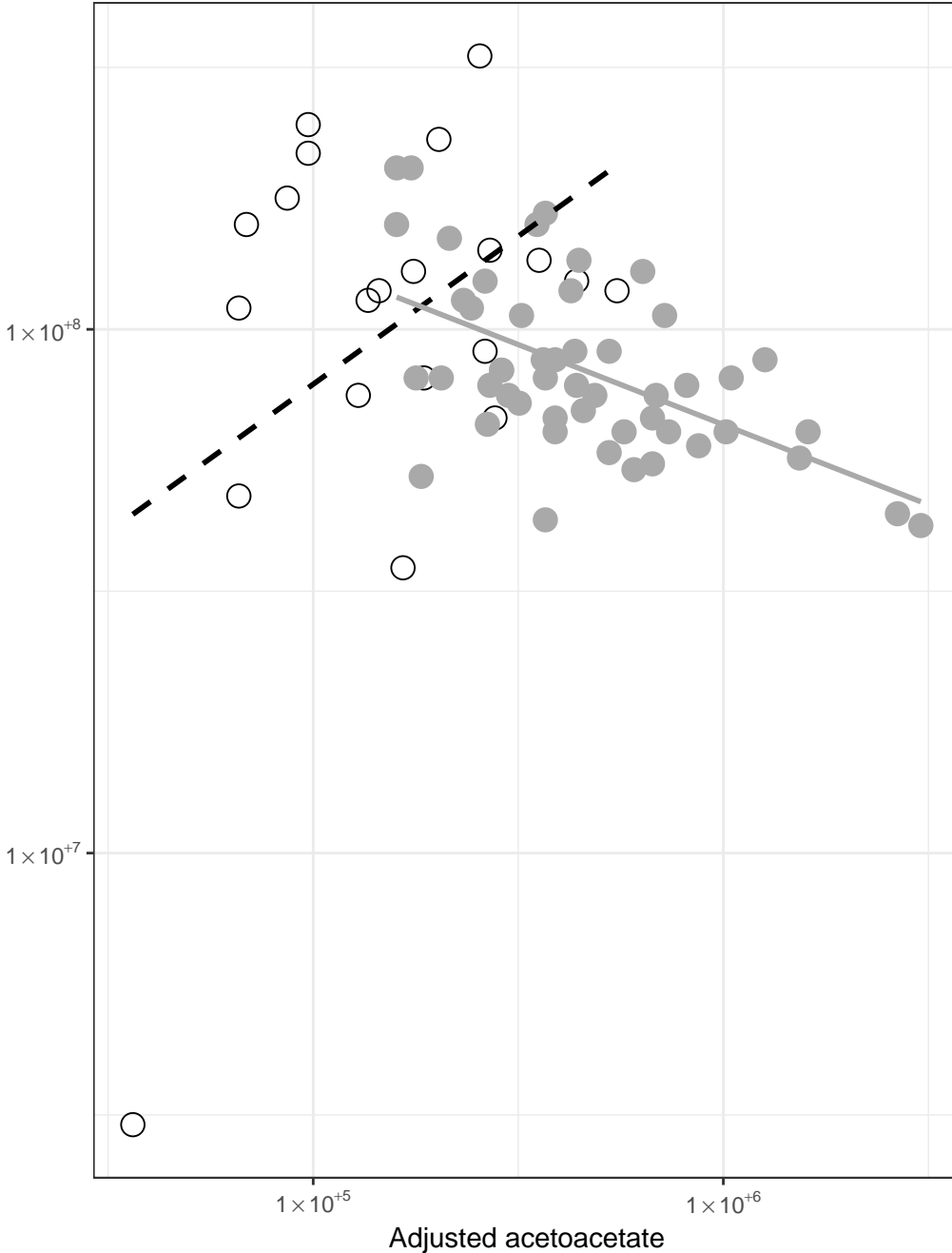

Supplement: S3 Fig — (PDF) [file pone.0224682.s006.pdf]
