# Supplementary material for: The plasma metabolome of women in early pregnancy differs from that of non-pregnant women
Source: PLoS One. 2019 Nov 14;14(11):e0224682. doi: 10.1371/journal.pone.0224682 (PMC6855901; doi:10.1371/journal.pone.0224682)
Supplement: S4 Fig — (PDF) [file pone.0224682.s007.pdf]

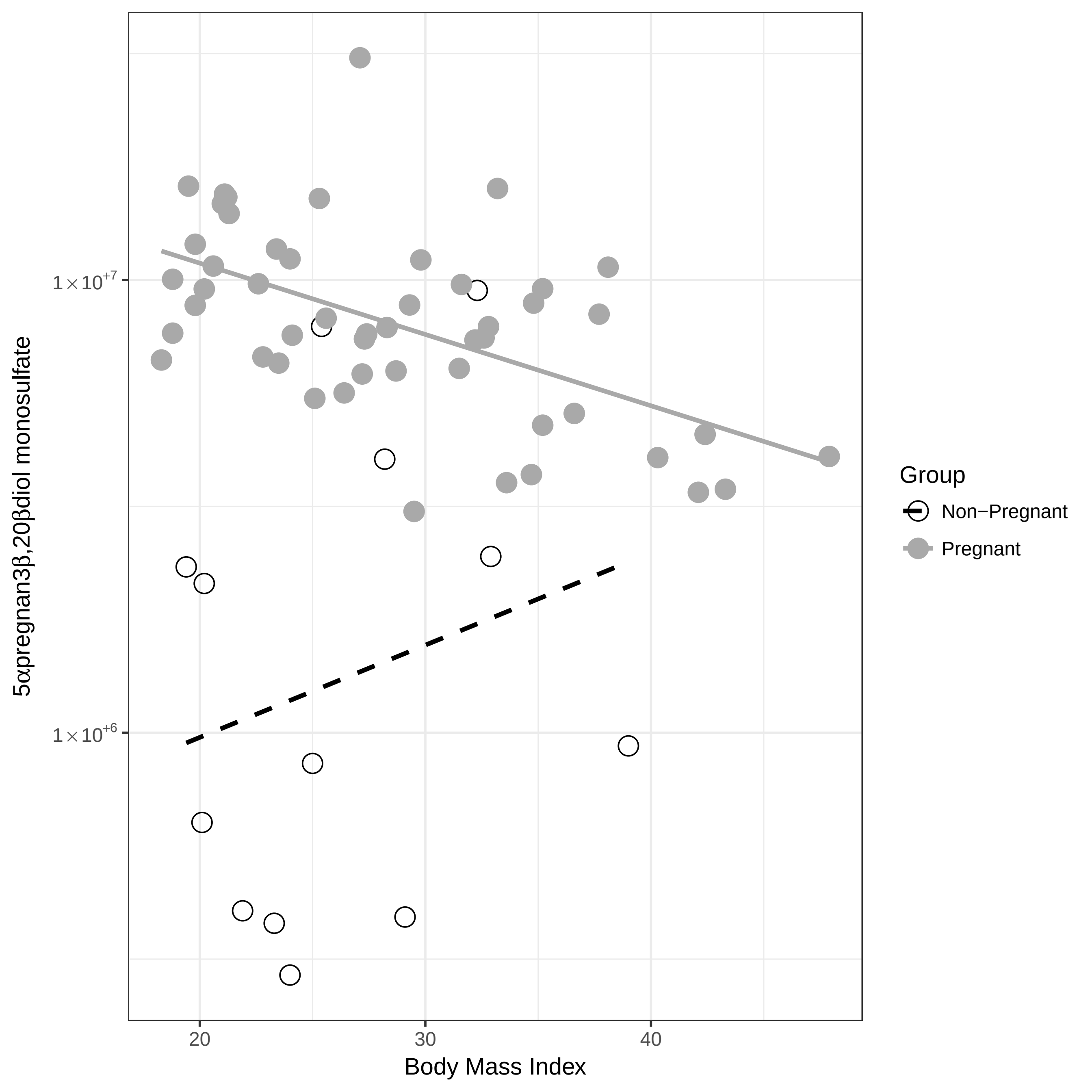

5αpregnan3β,20βdiol monosulfate
1×10^+7
1×10^+6
20
30
40
Body Mass Index
Group
Non−Pregnant
Pregnant
