# Supplementary material for: The plasma metabolome of women in early pregnancy differs from that of non-pregnant women
Source: PLoS One. 2019 Nov 14;14(11):e0224682. doi: 10.1371/journal.pone.0224682 (PMC6855901; doi:10.1371/journal.pone.0224682)

A)

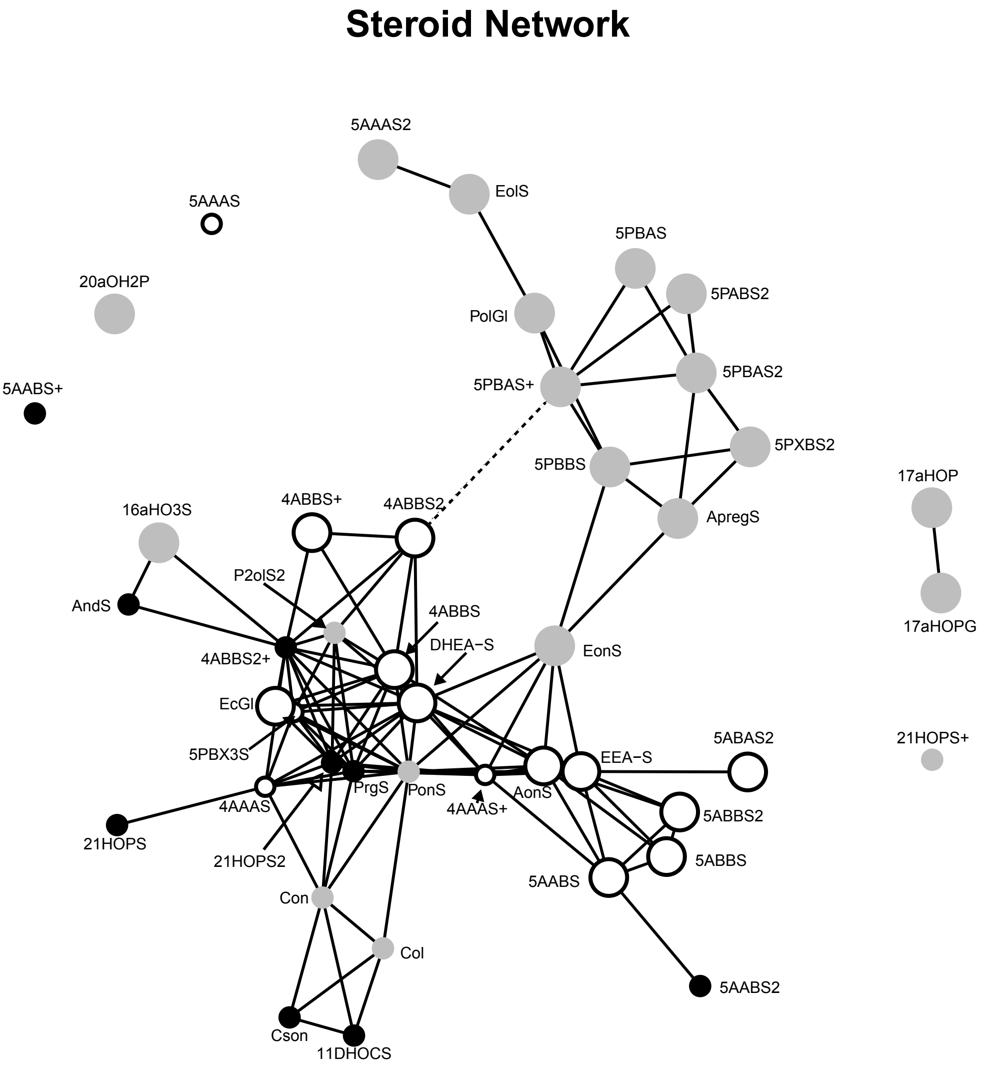
Steroid Network
5AAAS2
EolS
5AAAS
5PBAS
20aOH2P
5PABS2
PolGl
5AABS+
5PBAS+
5PBAS2
5PXBS2
5PBBS
17aHOP
16aHO3S
4ABBS+
4ABBS2
ApregS
P2olS2
AndS
4ABBS
17aHOPG
DHEA−S
EonS
4ABBS2+
EcGl
5PBX3S
EEA−S
5ABAS2
21HOPS+
PrgS
PonS
AonS
4AAAS
4AAAS+
5ABBS2
21HOPS
21HOPS2
5ABBS
5AABS
Con
Col
5AABS2
Cson
11DHOCS

B)

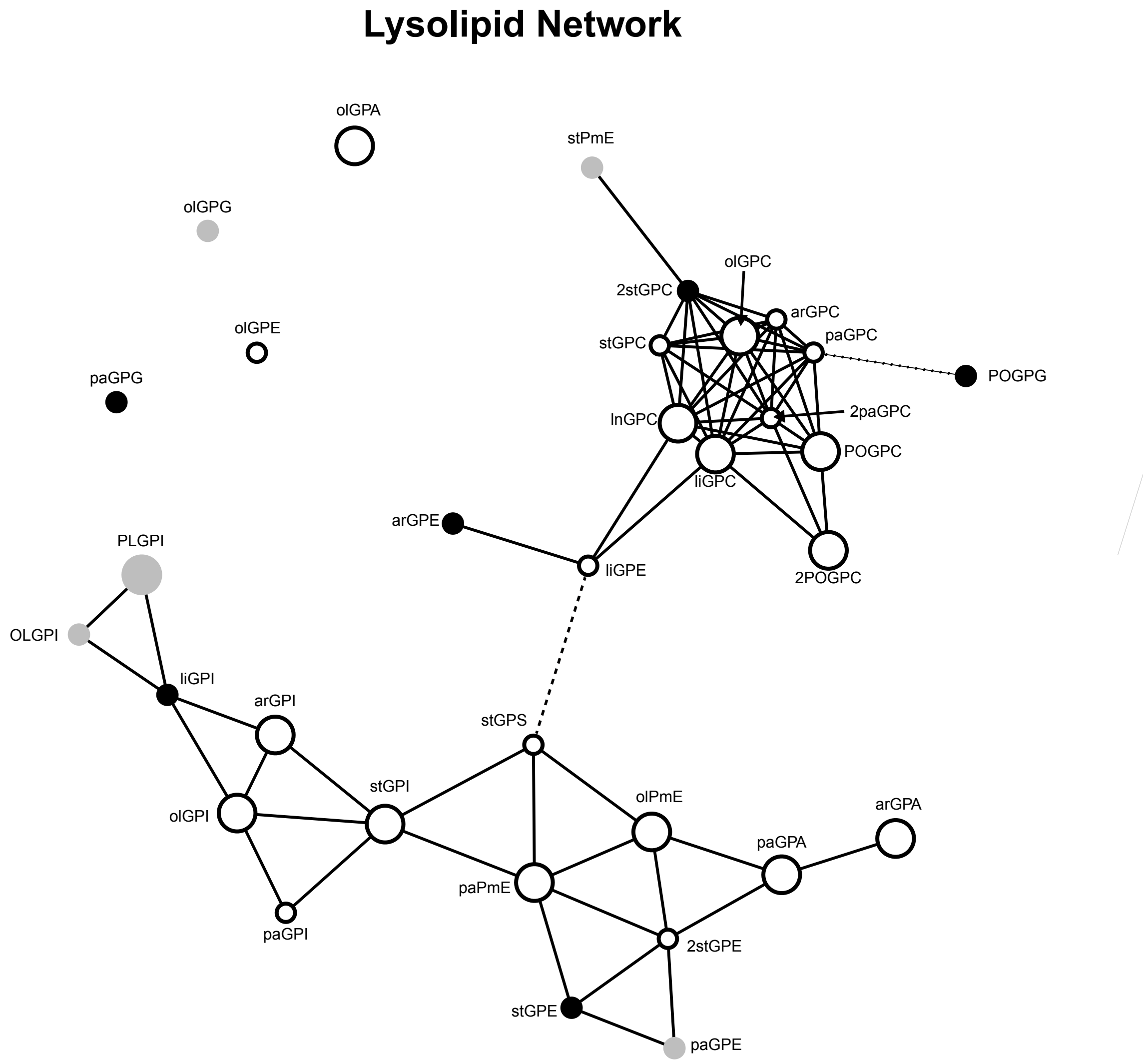

Lysolipid Network
olGPA
stPmE
olGPG
olGPC
2stGPC
arGPC
paGPC
stGPC
olGPE
POGPG
paGPG
2paGPC
lnGPC
POGPC
liGPC
arGPE
liGPE
2POGPC
PLGPI
OLGPI
liGPI
arGPI
stGPS
stGPI
olPmE
arGPA
olGPI
paGPA
paPmE
paGPI
2stGPE
stGPE
paGPE

Dipeptide Network

C)

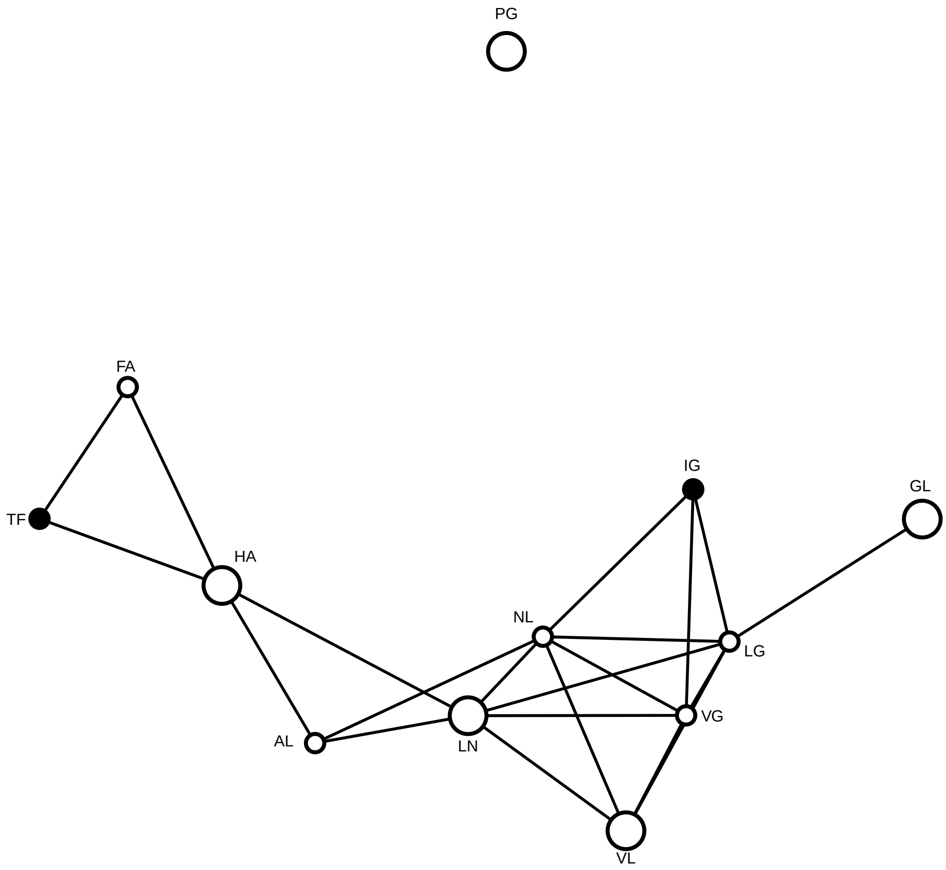

Bradykinin Network

D)

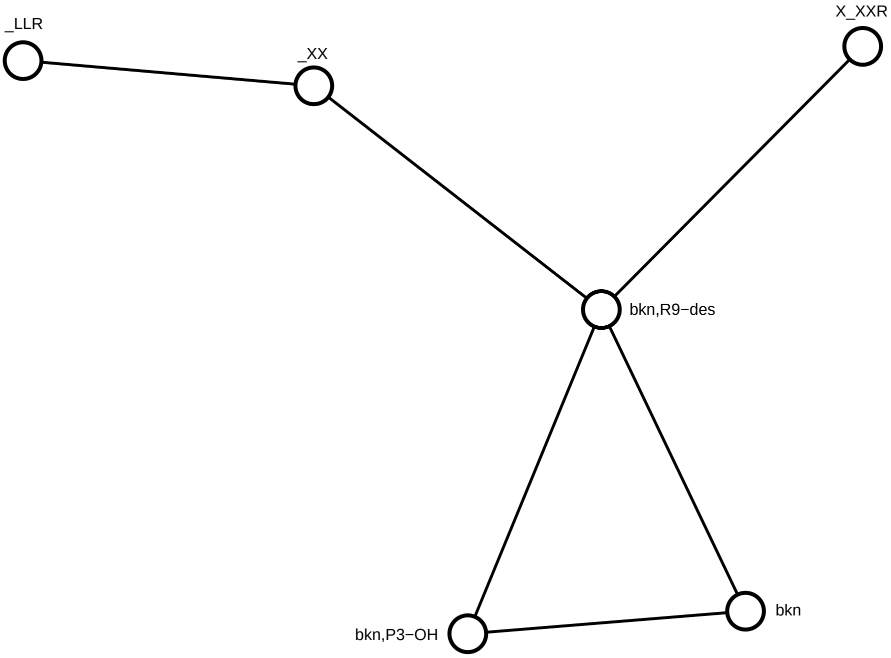

Xanthines (caffeine) Network

E)

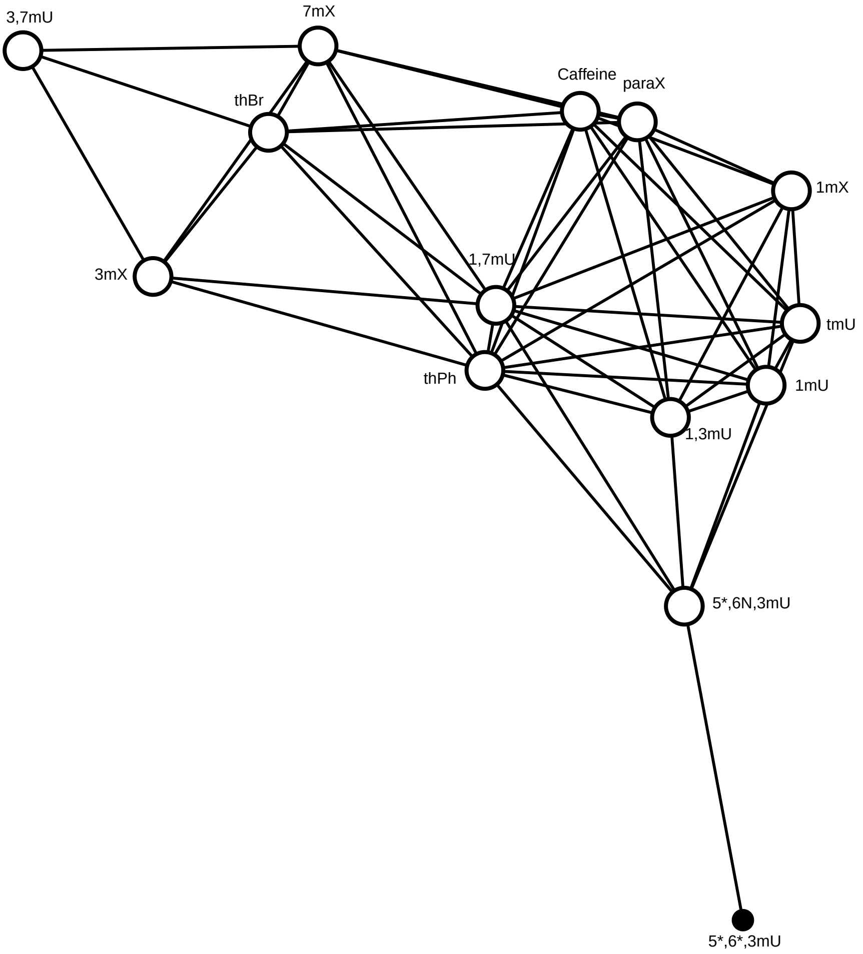

Supplement: S5 Fig — (PDF) [file pone.0224682.s008.pdf]
